# Principles of Carbon Catabolite Repression in the Rice Blast Fungus: Tps1, Nmr1-3, and a MATE–Family Pump Regulate Glucose Metabolism during Infection

**DOI:** 10.1371/journal.pgen.1002673

**Published:** 2012-05-03

**Authors:** Jessie Fernandez, Janet D. Wright, David Hartline, Cristian F. Quispe, Nandakumar Madayiputhiya, Richard A. Wilson

**Affiliations:** 1 Department of Plant Pathology, University of Nebraska–Lincoln, Lincoln, Nebraska, United States of America; 2 Proteomic and Metabolomic Core Facility, Redox Biology Center, Department of Biochemistry, University of Nebraska–Lincoln, Lincoln, Nebraska, United States of America; University of Melbourne, Australia

## Abstract

Understanding the genetic pathways that regulate how pathogenic fungi respond to their environment is paramount to developing effective mitigation strategies against disease. Carbon catabolite repression (CCR) is a global regulatory mechanism found in a wide range of microbial organisms that ensures the preferential utilization of glucose over less favourable carbon sources, but little is known about the components of CCR in filamentous fungi. Here we report three new mediators of CCR in the devastating rice blast fungus *Magnaporthe oryzae*: the sugar sensor Tps1, the Nmr1-3 inhibitor proteins, and the multidrug and toxin extrusion (MATE)–family pump, Mdt1. Using simple plate tests coupled with transcriptional analysis, we show that Tps1, in response to glucose-6-phosphate sensing, triggers CCR via the inactivation of Nmr1-3. In addition, by dissecting the CCR pathway using *Agrobacterium tumefaciens*-mediated mutagenesis, we also show that Mdt1 is an additional and previously unknown regulator of glucose metabolism. Mdt1 regulates glucose assimilation downstream of Tps1 and is necessary for nutrient utilization, sporulation, and pathogenicity. This is the first functional characterization of a MATE–family protein in filamentous fungi and the first description of a MATE protein in genetic regulation or plant pathogenicity. Perturbing CCR in Δ*tps1* and *MDT1* disruption strains thus results in physiological defects that impact pathogenesis, possibly through the early expression of cell wall–degrading enzymes. Taken together, the importance of discovering three new regulators of carbon metabolism lies in understanding how *M. oryzae* and other pathogenic fungi respond to nutrient availability and control development during infection.

## Author Summary

To succeed as pathogens, fungi such as the rice blast fungus *M. oryzae* must adapt their metabolism to nutrient availability within the host, but little is known about the genetic regulatory mechanisms involved. *M. oryzae* destroys enough rice to feed 60 million people annually, and understanding how the infection process is controlled would afford new targets for anti-rice blast strategies and shed light on regulatory pathways common to other pathogenic fungi. Here we use *M. oryzae* to identify and describe three new regulators of global carbon metabolism in filamentous fungi: the sugar-sensor Tps1; the transcription factor inhibitor proteins Nmr1-3; and a transmembrane efflux pump Mdt1 (the first pump of its type to be described in pathogenic filamentous fungi), which is essential for sporulation and pathogenicity. Tps1, Nmr1-3, and Mdt1 are shown to control the fungal response to glucose availability, and perturbation of this regulatory pathway abolishes disease. This work gives fresh insights into nutrient adaptation and the control of fungal development during infection and is thus applicable to a wide range of fungal pathogens.

## Introduction

**Figure 1:**
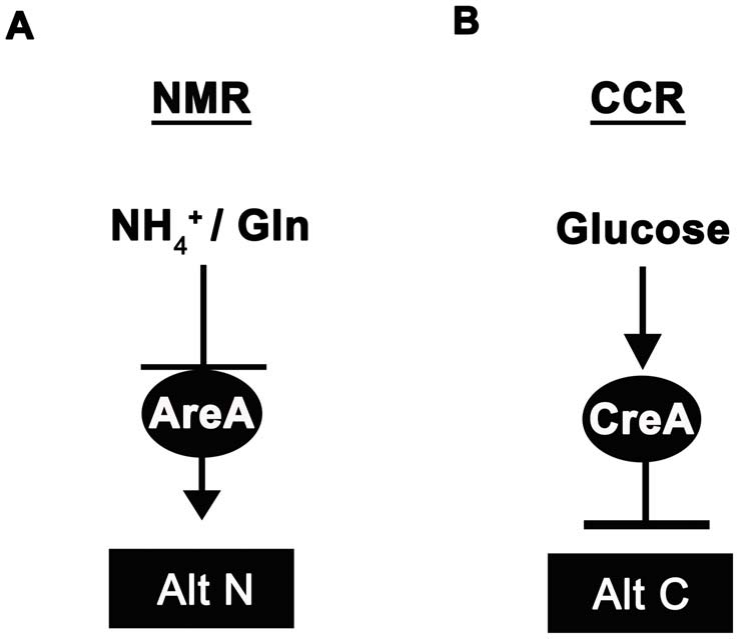
Ammonium and glucose are preferred nitrogen and carbon sources in filamentous fungi. (A) Nitrogen metabolite repression (NMR) ensures the preferential utilization of ammonium (NH_4_^+^) or L-glutamine as nitrogen sources by modulating the activity of a GATA family transcriptional activator (AreA in *Aspergillus nidulans*) necessary for the expression of genes involved in assimilating and utilizing alternative nitrogen sources (Alt N). (B) In the presence of glucose, carbon catabolite repression (CCR) acts via a negatively-acting transcriptional repressor (CreA in *Aspergillus nidulans*) to prevent the expression of genes required for utilizing alternative carbon sources (Alt C).

Fungi cause recalcitrant diseases of humans, animals and plants. In order to survive in environments with limited and variable resources, they have developed elegant and efficient genetic regulatory systems to enable them to respond rapidly to fluctuating nutritional conditions, but little is known about the components of these metabolic control pathways in multicellular fungal pathogens. Carbon and nitrogen metabolic regulation has, however, been extensively studied in model filamentous fungi such as the bread mold *Neurospora crassa* [1] and the soil saprophyte *Aspergillus nidulans* [2]–[8]. *A. nidulans* uses pathway specific gene induction to metabolize a wide range of carbon and nitrogen compounds, but this voracity is tempered by two global regulatory systems that ensure the preferential utilization of a few favoured carbon and nitrogen sources. The positive-acting GATA family transcription factor AreA functions in global nitrogen metabolite repression (NMR) to allow utilization of the most preferred nitrogen sources ammonium (NH_4_^+^) and L-glutamine (Figure 1A; reviewed in [7] and [8]). In the presence of NH_4_^+^or L-glutamine, the inhibitor protein NmrA [9] interacts with AreA to prevent nitrogen catabolic gene expression, but in the presence of less-preferred nitrogen sources such as nitrate (NO_3_^−^), NmrA dissociates from AreA, allowing it to activate the expression of more than 100 genes involved in alternative nitrogen source usage [7]. Carbon catabolite repression (CCR) on the other hand, operates via the negatively-acting zinc finger repressor CreA [4], [6], [10], [11] to ensure glucose is utilized preferentially by preventing the expression of genes required for the metabolism of less preferred carbon sources (Figure 1B).

Interestingly, both CCR and NMR regulatory systems converge on genes required for metabolizing a few key compounds that can be used as both carbon and nitrogen sources. For example, *A. nidulans* utilizes proline as both a carbon and nitrogen source [2], [3], [12], [13]. Dual CCR/NMR control of proline utilization ensures proline can be used as a nitrogen source in the presence of a repressing carbon source, and can be used as a carbon source in the presence of a repressing nitrogen source. Moreover, strains carrying *areA* loss-of-function mutations (*areA* ^−^) are unable to utilize proline as a source of nitrogen if a repressing carbon source (e.g. glucose) is present, but grow on proline in the presence of non-repressing carbon sources [2], [3]. Thus AreA is only required for the expression of proline structural genes in the presence of an active CreA protein. Loss of growth of *areA* ^−^ strains on glucose+proline media has been used as a selection to generate revertants of *areA* ^−^, restored for growth on this media, that result from mutations in CreA and the inactivation of CCR [3].

Like other fungal pathogens, the filamentous fungus *Magnaporthe oryzae*, cause of the devastating rice blast disease [14], [15], also faces challenges of nutrient limitation and variability but in a significantly different environment to that of *A. nidulans*. Rice blast disease is a grave threat to global food security [16] and results in 10–30% crop loss annually [17], although in some regions destruction of rice can reach 100%. The life cycle of *M. oryzae* begins when a three-celled conidium lands on the surface of the leaf and germinates [15]. In a nutrient-free and hydrophobic environment (ie. the leaf surface), the germtube swells and forms the dome-shaped infectious cell called the appressorium. Enormous turgor in the appressorium, formed from the accumulation of glycerol, acts on a thin penetration peg emerging from the base of the cell, forcing it through the surface of the leaf. However, this “brute-force” entry mechanism belies the fact that once within the host cell, the fungus spreads undetected from cell to cell in a biotrophic growth phase, extracting nutrients from the host in a manner that does not immediately kill the plant cell [18], [19]. Only after 72 hrs does *M. oryzae* enter its necrotic phase, forming characteristic lesions on the surface of the leaf from which aerial hyphae release spores to continue the infection process. During the infection cycle, global regulatory systems in *M. oryzae* must cope temporally with acquiring nutrients by stealth during biotrophy and by absorption during necrotrophy; and must respond spatially to the fluctuations in nutrient quality and quantity encountered throughout the host leaf. Moreover, plate tests show *M. oryzae* can grow on a wide range of carbon and nitrogen sources likely controlled by NMR and CCR ([20], [21]; Quispe and Wilson, unpublished data).

Although an AreA homologue, Nut1, has been characterized in *M. oryzae* and is not required for virulence [22], [23], no global regulators of carbon metabolism have been characterized in this fungus. In addition, little is known about CCR in other fungal pathogens, although overexpressing the *CREA* homologue in the plant pathogen *Alternaria citri* results in severe symptoms of black rot in citrus fruit [24]; CCR has been shown to be involved in isocitrate lyase and cell wall degrading enzyme production in the tomato pathogen *Fusarium oxysporum* [25]; and the absence of either hexokinase or glucokinase protein in the human pathogen *Aspergillus fumigatus* results in loss of CCR and the induction of isocitrate lyase activity in the presence of glucose [26]. Recently, trehalose-6-phosphate synthase (Tps1) has emerged as a glucose-6-phosphate (G6P) sensor that, *inter alia*, integrates carbon and nitrogen metabolism to regulate infection by *M. oryzae* [21], [23]. Tps1 controls infection-related gene expression via a novel NADPH-dependent genetic switch. In response to G6P, Tps1 activates glucose-6-phosphate dehydrogenase, leading to the elevated production of the reduced dinucleotide NADPH from NADP and G6P. As NADPH levels increase at the expense of NADP, three *M. oryzae* homologues of the NmrA inhibitor protein- Nmr1, Nmr2 and Nmr3 - become inactivated, resulting in the activation of at least three GATA factors (including Nut1) and the expression of genes required for pathogenicity (Figure S1).

We undertook this study to determine whether G6P sensing by Tps1 in filamentous fungi regulates carbon metabolism via CCR, to identify what proteins constitute CCR, and to understand how CCR impacts pathogenicity - processes currently unknown in *M. oryzae* and little understood in other fungi [10], [11]. Here we show for the first time in filamentous fungi that the G6P sensor for triggering CCR is Tps1. We show in *M. oryzae* how Tps1 regulation of CCR involves Nmr1-3, and how the modulation of CCR by the Nmr1-3 inhibitor proteins occurs independently of Nut1 - thus revealing a hitherto unrecognized role for Nmr-like proteins in carbon regulation. Δ*nut1* strains, like *areA* ^−^ strains, are unable to grow on proline in the presence of glucose. To identify additional components of CCR and to characterize their role in pathogenicity, we used *Agrobacterium tumefaciens-* mediated mutagenesis to target CCR by selecting for Δ*nut1* strains restored in their ability to grow on media containing glucose and proline. In this manner we identified a MATE-family efflux pump [27], Mdt1, as an additional regulator of CCR. Characterization of mutants disrupted in the *MDT1* gene showed they were misregulated for carbon metabolism even in the presence of glucose. They were also severely attenuated in sporulation and, although they could form appressoria and were not sensitive to reactive oxygen species (ROS), they were unable to cause disease. Therefore, we demonstrate Mdt1 is essential for nutrient adaptability and pathogenicity in *M. oryzae*. *In toto*, this work describes three new classes of global carbon metabolic regulators in filamentous fungi; it is the first study to characterize a MATE-family efflux pump in filamentous and plant pathogenic fungi; and is the first study to assign a regulatory function to a MATE protein in any organism.

## Results/Discussion

### Genes for metabolizing compounds that are both carbon and nitrogen sources are subject to CCR and NMR in *M. oryzae*

**Figure 2:**
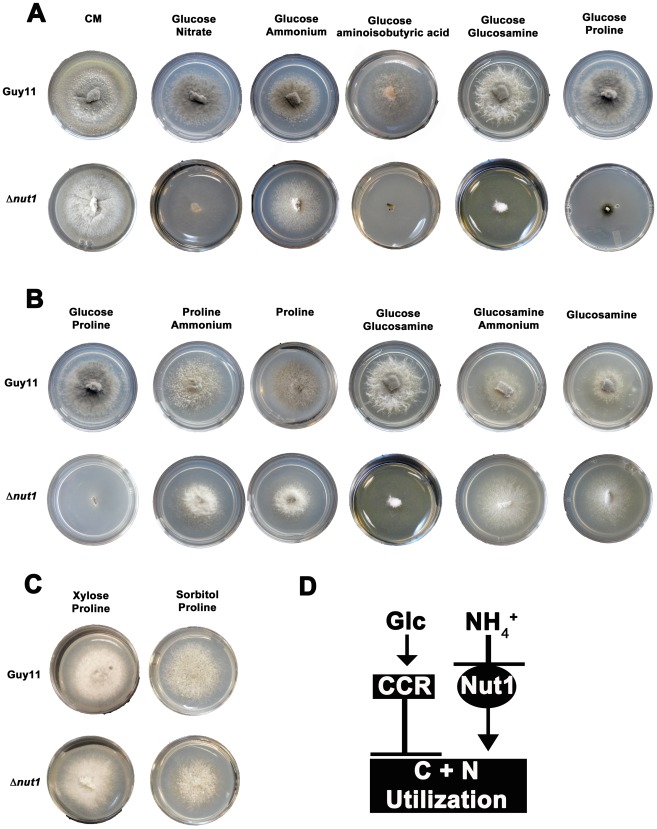
Compounds that are both carbon and nitrogen sources are subject to CCR and NMR. (A) Plate tests of nitrogen utilization by Guy11 and Δ*nut1* strains in the presence of glucose. Strains were grown for 10 days on complete media (CM) or minimal media containing 10 mM glucose supplemented with 10 mM of the appropriate compound. Because Δ*nut1* strains are defective for nitrogen metabolite repression, they can grow on ammonium (NH_4_^+^) as nitrogen source but cannot grow on alternative nitrogen sources that require a functional Nut1 protein, such as nitrate (NO_3_^−^). In addition, Δ*nut1* strains cannot grow on aminoisobutyric acid, glucosamine and proline as sole nitrogen sources in the presence of glucose, demonstrating these compounds require an active Nut1 protein for utilization as nitrogen sources. (B) Both Guy11 and Δ*nut1* strains can metabolize glucosamine and proline as sole carbon sources in the presence or absence of a repressing nitrogen source, suggesting the utilization of these compounds as carbon sources is subject to CCR. (C) Replacement of glucose with the derepressing (i.e. CCR inactivating) carbon sources xylose or sorbitol fully restores the ability of Δ*nut1* strains to use proline as a nitrogen source, confirming these genes are under dual CCR and NMR. (D) Taken together, the expression of genes required for metabolizing compounds that are both nitrogen and carbon sources (represented by the box labeled C+N utilization) are subject to both CCR and NMR. Glc is glucose. CCR represents a signal transduction pathway of unknown components leading to glucose repression. Nut1 is the Nut1 protein.

This study began with an interest in understanding how the metabolism of compounds having the potential to be both carbon and nitrogen sources are regulated in *M. oryzae*. Our initial investigations found that Δ*nut1* strains generated by Wilson et al. in a previous study [23] could not utilize three such compounds - aminoisobutyric acid, proline and glucosamine – in the presence of glucose compared to the wild type Guy11 strain. The inability of Δ*nut1* strains to grow on proline as a nitrogen source contradicts an earlier study by Froeliger and Carpenter, where deletion of *NUT1* was shown to allow growth on proline [22]. We therefore independently generated new Δ*nut1* strains (Figure 2A) and verified that they also cannot grow on proline, in addition to aminoisobutyric acid and glucosamine, in the presence of glucose. This suggests the metabolism of proline, glucosamine and aminoisobutyric acid requires an active Nut1 protein for utilization as nitrogen sources when glucose is present (Figure 2A).

Other than an inability to use proline as a nitrogen source, in all other aspects, our Δ*nut1* strains have the same phenotype as that reported by Froeliger and Carpenter [22]. This includes an inability to grow on defined minimal media containing nitrate (NO_3_^−^) or nitrite as sole nitrogen sources (Figure S2A); good growth on ammonium (NH_4_^+^), glutamate and alanine as sole nitrogen sources (Figure S2A); and small lesion sizes on host leaf [23]. We cannot explain this discrepancy, but in light of the analyses that follow, we conclude deleting *NUT1* abolishes proline utilization in the presence of glucose.

We next determined that the wild type strain, Guy11, could not utilize aminoisobutyric acid as a carbon source (Figure S2B). This compound is therefore not both a carbon and nitrogen source for *M. oryzae*, and was excluded from further analysis. Focusing on glucosamine and proline, we found that although unable to use these compounds as sole nitrogen sources in the presence of glucose, Δ*nut1* strains, like Guy11, utilized these compounds as carbon sources in the absence of glucose - both in the presence and absence of a repressing nitrogen source (NH_4_^+^) (Figure 2B). This suggests these compounds do not require an active Nut1 when metabolized as a carbon source and are therefore under CCR control. In addition, Δ*nut1* strains were restored for growth on proline as a nitrogen source in the presence of the derepressing carbon sources xylose and sorbitol (Figure 2C), confirming the metabolism of these compounds is subject to both CCR and nitrogen metabolite repression. We conclude that an active Nut1 protein is required for using these dual compounds as nitrogen sources in the presence of glucose (ie. when CCR is active), but is not required in the absence of glucose or in the presence of derepressing carbon sources (ie. when CCR is inactive) (Figure 2D).

### G6P sensing by Tps1 is required to activate CCR

**Figure 3:**
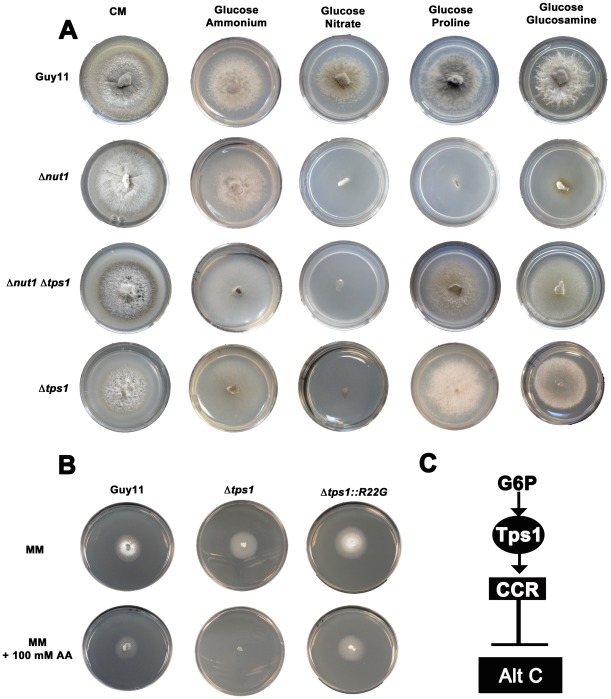
Tps1 regulates CCR in response to G6P sensing. (A) CCR is Tps1 dependent. Strains were grown for 10 days on CM or minimal media supplemented with 10 mM of the appropriate carbon and nitrogen source. Like Δ*nut1*, Δ*tps1* and Δ*tps1 Δnut1* strains are unable to utilize nitrate as nitrogen source. However, deleting the *TPS1* gene in Δ*nut1* strains restores growth on proline and glucosamine as nitrogen sources, demonstrating that CCR is inactivated in Δ*tps1*- carrying strains. (B) G6P sensing by Tps1 activates CCR. To mitigate against AA evaporation, best results were obtained when Guy11, Δ*tps1* and Δ*tps1::R22G* strains were grown for 5 days on 85 mm petri dishes containing either glucose-rich minimal media with 55 mM glucose and 10 mM NH_4_^+^ as sole carbon and nitrogen sources, respectively, or the same medium supplemented with 100 mM of the toxic analogue allyl alcohol (AA). Δ*tps1* strains were sensitive to 100 mM allyl alcohol, indicating they are carbon derepressed (i.e. CCR is inactivated) in the presence of glucose. Like Guy11, Δ*tps1::R22G* strains were not sensitive to 100 mM allyl alcohol, suggesting CCR operates correctly in the Δ*tps1::R22G* G6P sensing strains. (C) G6P sensing by Tps1 is the trigger for CCR resulting in the inhibition of alternative carbon source (Alt C) utilization by *M. oryzae*.

The above results suggested that CCR plays an active regulatory role in *M. oryzae* carbon metabolism. We continued our characterization of carbon metabolism in the rice blast fungus by determining what role, if any, Tps1 might play in carbon regulation. Tps1 is a G6P sensor that integrates carbon and nitrogen metabolism and is essential for pathogenicity. In response to G6P, Tps1 modulates NADPH levels to inactivate the Nmr1-3 inhibitor proteins and activate transcription factors including Nut1 [23]. Thus, Δ*tps1* mutants cannot grow on nitrate as nitrogen source because the Nmr1-3 inhibitor proteins constitutively inactivate Nut1 in this strain [21], [23], [28]. Δ*tps1* strains are also affected in glycogen metabolism [21], suggesting Tps1 might regulate carbon metabolism. To determine how extensive Tps1-dependent carbon regulation might be, we generated a Δ*tps1* Δ*nut1* double mutant and showed that, unlike the Δ*nut1* single mutant, it can utilize proline and glucosamine as nitrogen sources in the presence of glucose (Figure 3A). This suggests CCR, at least for proline and glucosamine metabolism, is Tps1-dependent.

In addition to compounds that are both carbon and nitrogen sources, might Tps1-dependent CCR also regulate the metabolism of compounds that are carbon sources only? In the presence of glucose, CCR is known to inhibit the expression of genes encoding alcohol dehydrogenases that convert alcohols into acetyl-coA. Allyl alcohol is used as an assay for carbon derepression because it is converted by alcohol dehydrogenase to the very toxic compound acrylaldehyde. Wild type *M. oryzae* strains are resistant to allyl alcohol when grown on repressing carbon sources (i.e. glucose) but inactivation of CCR by derepressing carbon sources renders *M. oryzae* sensitive to allyl alcohol [20]. Mutations that inactivate CCR should also result in carbon derepression and sensitivity to allyl alcohol in the presence of glucose. In our study, Δ*tps1* mutant strains were grown on a glucose-rich minimal media containing 55 mM glucose (ie. 1% glucose) with 10 mM NH_4_^+^ as sole carbon and nitrogen source, respectively, with or without 100 mM allyl alcohol (AA). Figure 3B and Figure S3A show that, compared to Guy11, ally alcohol was extremely toxic to Δ*tps1* strains at this concentration, suggesting Δ*tps1* strains were strongly derepressed for alcohol metabolism in the presence of glucose. This indicates Tps1 controls CCR to regulate, in addition to proline and glucosamine, broad aspects of carbon metabolism in response to glucose.

We next asked whether regulation of CCR by Tps1 occurs via G6P sensing. G6P and UDP-glucose are native substrates for Tps1. Previous work showed Tps1 proteins carrying the amino acid substitutions R22G or Y99V in the G6P binding pocket were abolished for trehalose-6-phosphate production but could still sense G6P and were pathogenic, thus demonstrating a sugar signaling role for Tps1 independent of its biosynthetic function [21]. We found that compared to Δ*tps1* strains, strains carrying the constructs Δ*tps1::R22G* (Figure 3B and Figure S3A) and Δ*tps1::Y99V* (Figure S3A) - encoding the R22G and Y99V substitutions in Tps1, respectively - were insensitive to 100 mM AA in the presence of glucose and, unlike the Δ*tps1* parental strains, were not inactivated for CCR. Thus, G6P sensing by Tps1 is required for CCR.

In *Saccharomyces cerevisiae*, phosphorylation of glucose and fructose by the hexokinase protein Hxk2p results in CCR [29]. In addition, Hxk2p regulates CCR independently of hexose phosphorylation because mutant Hxk2p proteins with reduced catalytic activity still demonstrate some glucose repression, suggesting Hxk2p might induce CCR via a non-metabolic process likely requiring nuclear localization [30]. *Magnaporthe oryzae* carries genes encoding two putative hexokinases (*HXK1* and *HXK2*) and one glucokinase (*GLK1*). Δ*hxk1* [21] and Δ*glk1* [31] gene deletion strains are fully pathogenic, but the role of these genes in CCR has not been examined. To determine if *Magnaporthe* hexose kinase proteins have a non-metabolic role in CCR upstream of Tps1 in the G6P signaling pathway, we deleted *GLK1*, *HXK1* and the previously uncharacterized *HXK2* gene from the Guy11 genome by homologues gene replacement [23] and tested the resulting deletion strains for loss of CCR. Figure S3B shows that neither hexose kinase deletion strain demonstrated susceptibility to 100 mM allyl alcohol in the presence of 55 mM glucose, suggesting CCR is still operating in these deletion strains. Thus, unlike yeast but similar to *A. nidulans* [11], loss of the hexokinase or glucokinase proteins in *Magnaporthe* does not affect CCR. However, multiple hexose kinase deletion mutants would be expected to be inactive for CCR in the presence of glucose by virtue of their inability to form G6P, the trigger for CCR. The generation and analysis of multiple hexose kinase gene deletion strains is a future goal of our research.

Taken together, these results suggest G6P sensing by Tps1 is the key step in the regulation of CCR in *Magnaporthe* (Figure 3C), and is the first report of how G6P triggers CCR in filamentous fungi.

### Transcriptional studies, plate growth tests, and proteomic analysis reveal Tps1 regulates glucose metabolism and suppresses alternative carbon source utilization

To understand how Tps1-dependent CCR might regulate carbon metabolism, we used quantitative real time PCR (qPCR) to analyze the expression of genes required for glucose metabolism and alternative carbon source utilization in Guy11, compared to Δ*tps1* strains, following growth on minimal media containing glucose and NH_4_^+^. Nitrogen-repressing media was chosen to eliminate a role for Nut1 in the expression of these genes (see below), but similar fold changes were also seen when the strains were grown on NO_3_^−^ minimal media (Figure S4). Strains were grown in complete media (CM) for 48 hr before switching to minimal media containing 55 mM glucose with 10 mM NH_4_^+^ or 10 mM NO_3_^−^ as sole nitrogen sources for 16 hr (following [23]). CM is used as the initial growth condition in *Magnaporthe* switch experiments because when fresh CM is added at 24 hr, it allows strong mycelial growth of *Magnaporthe* strains without resulting in the rapid melanization of mycelia observed for growth in minimal media. Similarly, mycelia was switched to minimal media for 16 hr to allow maximum gene induction while avoiding the melanization of mycelia that occurs after this time.

**Figure 4:**
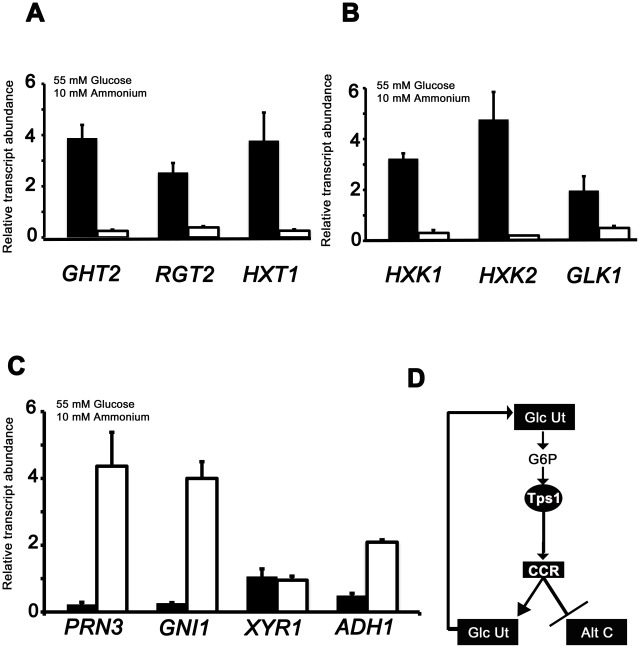
qPCR analysis of Tps1-dependent gene expression. The expression of carbon metabolizing genes were analyzed in strains of Guy11 (black bars) and Δ*tps1* (open bars) that were grown in CM media for 48 hr before switching to 55 mM glucose+10 mM NH_4_^+^ minimal media for 16 hr. Gene expression results were normalized against expression of the ß-tubulin gene (*TUB2*). Results are the average of at least three independent replicates, and error bars are the standard deviation. (A) The expression of three genes encoding putative glucose transporters, *GHT2*, *RGT2* and *HXT1*, is Tps1-dependent. (B) qPCR analysis of hexose kinase gene expression in Guy11 and Δ*tps1* strains shows that *HXK1*, *HXK2* and *GLK1* expression is Tps1-dependent. (C) Tps1 is required for repressing proline (*PRN3*) glucosamine (*GNI1*) and alcohol (*ADH1*) metabolic gene expression during growth on glucose-containing media. (D) Based on plate tests and transcriptional data, we propose available glucose is taken up into the cell and phosphorylated to G6P by hexose transporters and hexose kinases, collectively termed glucose utilization processes (Glc Ut). In response to G6P sensing, Tps1 activates CCR, leading to the repression of genes required for alternative carbon source utilization (Alt C) and the expression of genes required for glucose utilization (Glc Ut), which would in turn increase the availability of G6P in the cell.

By sequence homology to known glucose transporters in yeast, we studied the expression of genes encoding two putative high affinity glucose transporters (*GHT2* and *RGT2*), and one putative low affinity glucose transporter (*HXT1*) (Figure 4A; Table S1). We also studied the expression of hexose kinase genes likely involved in the first step of glucose metabolism: *HXK1*, *HXK2* and *GLK1* (Figure 4B, Table S1). Figure 4A and Figure 4B show that genes for importing and metabolizing glucose are reduced in expression in Δ*tps1* strains compared to Guy11 during growth on minimal media containing glucose.

The differences in gene expression of glucose transport and metabolism genes in Guy11 or Δ*tps1* strains were similar regardless of nitrogen source (Figure 4 and Figure S4). One notable exception was *GHT2* that appeared to be elevated in Δ*tps1* strains during growth on NO_3_^−^ media (Figure S4A) compared to Guy11. Because Δ*tps1* strains are unable to utilize nitrate, we considered that *GHT2* might be expressed in response to nitrogen starvation. To test this, we studied the expression of *GHT2* in the mycelia of Guy11 strains grown in NH_4_^+^ minimal medium with 55 mM glucose, or in 55 mM glucose minimal media lacking a nitrogen source. Figure S4C shows *GHT2* is elevated in Guy11 under nitrogen starvation conditions. Thus, a real lack of a metabolizable nitrogen source (in the case of Guy11 on nitrogen starvation media) or a perceived lack of nitrogen source (in the case of Δ*tps1* strains on nitrate media) induces *GHT2* expresssion, suggesting multiple nutritional signals converge on *GHT2*. Identifying what these signals might be warrants further analysis in the future.

We also examined the expression of four genes in Guy11 and Δ*tps1* strains necessary for alternative carbon source utilization following growth on 55 mM glucose and 10 mM NH_4_^+^minimal media: *PRN3* encoding a putative L-Δ^1^-pyrroline-5-carboxylate dehydrogenase likely required for proline utilization; *GNI1* encoding a putative glucosamine-6-phosphate isomerase/deaminase required for glucosamine metabolism; *XYR1* encoding a putative xylose reductase involved in xylose metabolism; and *ADH1* encoding a putative alcohol dehydrogenase (Figure 4C and 4D; Table S1). In contrast to glucose importing and metabolizing genes, the expression of genes for utilizing some alternative carbon sources (proline, glucosamine and alcohol but not xylose) are significantly elevated in Δ*tps1* during growth on glucose (*Student's t-test* p≤0.05).

The expression of *PRN3*, *GNI1* and *XYR1* following growth on nitrate media is shown in Figure S4D. The expression of *ADH1* following growth on nitrate media is shown in Figure S4E and is strongly up-regulated in Δ*tps1* strains.

The expression of a proline-metabolizing gene in Δ*tps1* in the absence of inducer might arise from internal proline carried over from the nutrient rich CM starter culture. To determine if this is the case, we repeated the mycelial switch experiment of Guy11 and Δ*tps1* but following 48 hr growth in CM, each strain was transferred to a starvation minimal media lacking both a source of glucose and nitrogen for 12 hr before switching into minimal media with 55 mM glucose and 10 mM NO_3_^−^ for 16 hr. The rationale is that internal sources of proline should be metabolized during growth under starvation conditions and would not be available to induce proline gene expression during growth in minimal media with a carbon and nitrogen source. Nonetheless, even when including a starvation shake condition, expression of *PRN3* was still significantly elevated in Δ*tps1* strains compared to Guy11 (*Student's t-test* p≤0.01; Figure S4F), suggesting derepression of at least one proline utilizing gene can occur in Δ*tps1* strains in the absence of an inducer.

Together with Figure 3B, we conclude that Tps1-mediated CCR, via G6P sensing, is required for the glucose-mediated induction of glucose utilization genes and the repression of genes required for metabolizing alternative carbon sources (Figure 4D).

**Figure 5:**
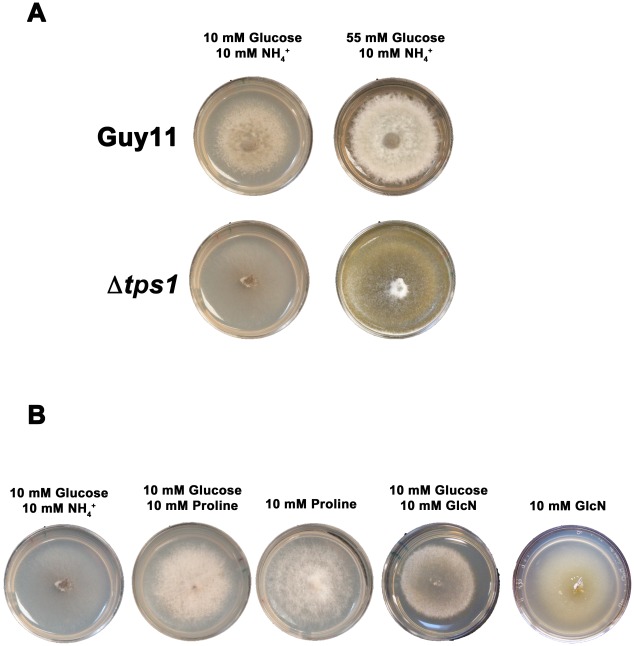
Glucose metabolism is impaired in Δ*tps1* strains. (A) Guy11 and Δ*tps1* strains were grown for 10 days on minimal media containing 10 mM NH_4_^+^ and either 10 mM or 55 mM (1%) glucose. Δ*tps1* strains grew better on 55 mM glucose. (B) Δ*tps1* strains were grown for 10 days on minimal media containing the indicated carbon and nitrogen sources. Better growth was obtained when an alternative carbon source to glucose – such as proline or glucosamine (GlcN) - was used.

Next, we considered how loss of CCR in Δ*tps1* strains affects fungal physiology. Figure 3C and the transcriptional results shown in Figure 4 and Figure S4 indicated Δ*tps1* strains should be impaired in glucose metabolism due to the inactivation of CCR in the presence of glucose and the resulting abherrant affect on glucose metabolizing gene expression. Altered glucose metabolism in Δ*tps1* strains compared to Guy11 is supported by two lines of evidence in Figure 3A. First, Figure 3A shows that Δ*tps1* and Δ*tps1* Δ*nut1* strains were reduced for growth on minimal media with 10 mM glucose and 10 mM NH_4_^+^ compared to the parental strains (Figure 3A). This is in contrast to previous reports that demonstrated strong growth of Δ*tps1* on ammonium minimal media [21], [23]. However, previous studies used 1% (ie 55 mM) glucose as carbon source, with nitrate or ammonium as nitrogen source, and Figure 5A shows that Δ*tps1* strains grew better on ammonium minimal media when high (55 mM) glucose concentrations were used compared to lower (10 mM) levels of glucose. Thus, Δ*tps1* strains grow poorly on low concentrations of glucose compared to Guy11. It should be noted that Δ*tps1* strains were not improved for growth on nitrate-media under any glucose conditons tested (up to 10% glucose, not shown), consistent with the hypothesis that Tps1 is required for integrating G6P availability, G6PDH activity and NADPH production during growth on nitrate [21].

A second piece of evidence for glucose metabolic defects of Δ*tps1* strains comes from the analysis of growth on proline and glucosamine containing minimal media. Figure 3A shows that growth of Δ*tps1* strains on 10 mM glucose+10 mM proline and 10 mM glucose+10 mM glucosamine minimal media was much weaker than in Guy11, but stronger than growth of Δ*tps1* strains on 10 mM glucose+10 mM NH_4_^+^. This suggested proline and glucosamine might be used as alternative but poorer sources of carbon for Δ*tps1* strains even in the presence of glucose. To test this, we looked at the growth of Δ*tps1* on proline and glucosamine as sole carbon and nitrogen sources. Figure 5B shows that compared to growth on 10 mM glucose+10 mM NH_4_^+^ media, Δ*tps1* strains grew stronger on media containing proline or glucosamine as a sole nitrogen source, a sole carbon source, or as both a carbon and nitrogen source. Taken together, deletion of Tps1 results in poor growth on glucose media compared to Guy11, which is partially remediated by alternative, less-preferred carbon sources such as proline and glucosamine.

**Figure 6:**
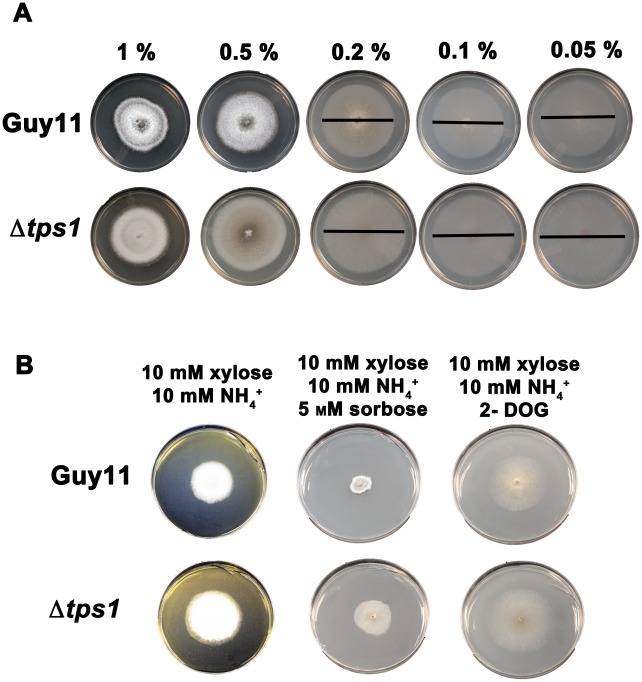
Glucose uptake and phosphorylation is not impaired in Δ*tps1* strains. (A) To determine if Δ*tps1* strains were defective in glucose uptake, Guy11 and Δ*tps1* were grown for 10 days on 85 mm petri-dishes containing minimal media with 10 mM NH_4_^+^ and glucose at final concentrations in the range of 1%–0.05% (indicated above the plates). Δ*tps1* radial growth was not reduced compared to Guy11 at low glucose concentrations. The diameters of sparsely growing colonies on low glucose media are indicated with a black bar for ease of viewing. (B) To determine if Δ*tps1* strains were defective in glucose uptake, Guy11 and Δ*tps1* were grown for 10 days on 85 mm petri-dishes containing carbon derepressing minimal media consisting of 10 mM xylose+10 mM NH_4_^+^ as sole carbon and nitrogen sources and the same media supplemented with 5 mM sorbose or 50 µg/mL 2-deoxyglucose (2-DOG). Δ*tps1* were not more resistant to sorbose or 2-DOG compared to Guy11, suggesting glucose uptake and phosphorylation is not significantly impaired in Δ*tps1* strains.

We next sought to determine whether Δ*tps1* strains were impaired in glucose metabolism due to defects in sugar uptake and phosphorylation or because they were unable to assimilate phosphorylated glucose. The sugar transport and hexose kinase expression data presented in Figure 4A and 4B suggested that reduced uptake and phosphorylation of glucose by Δ*tps1* strains might result in low internal G6P levels and the observed loss of CCR. Indeed, a class of carbon derepressed mutants of *A. nidulans* were found to result from defective glucose uptake [3]. However, several lines of evidence suggest Δ*tps1* strains are not reduced for glucose uptake and phosphorylation during growth on glucose-rich (55 mM) minimal media. Firstly, Wilson et al. [21] demonstrated that although G6PDH activity was reduced in Δ*tps1* strains during growth on nitrate compared to Guy11, hexokinase activity in Δ*tps1* strains was not affected, suggesting different mechanisms for hexokinase transcriptional and post-translational control that warrant further investigation in the future. Secondly, G6P levels are significantly elevated, not depleted, in the mycelia of Δ*tps1* strains under both NO_3_^−^ and NH_4_^+^ nitrogen regimes [21] suggesting G6P assimilation –via the pentose phoshate pathway - but not G6P production was impaired. Thirdly, although Δ*tps1* strains grow with reduced hyphal mass on minimal media with 10 mM glucose+10 mM NH_4_^+^ compared to Guy11 (Figure 5A), radial growth was not affected, again suggesting glucose assimilation but not uptake is impaired. Indeed, growth of glucose uptake mutants would be significantly inhibited on low glucose media, but the radial growth of Δ*tps1* strains on low glucose concentrations (0.2% to 0.05% glucose final concentration) was comparable to that of Guy11 on the same media (Figure 6A). This suggested that Δ*tps1* strains do not grow significantly different to Guy11 on carbon-limiting (ie glucose-derepressing) media, as would be expected if CCR was constitutively inactivated in Δ*tps1* strains. Finally, the carbon derepressed mutants of *A. nidulans* that were found to result from defective glucose uptake [3] were also resistant to both the toxic glucose analogue 2-deoxyglucose (2-DOG), which requires uptake and phosphorylation by hexokinase activity for toxicity, and the toxic sugar sorbose [32] during growth under carbon derepressing conditions. When grown on carbon derepressing minimal media comprising 55 mM xylose and 10 mM NH_4_^+^ as sole carbon and nitrogen sources, we observed, however, that disruption of Δ*tps1* did not confer resistance to these toxic analogues (Figure 6B). Taken together, these four lines of evidence indicated uptake and phosphorylation of glucose was not greatly impaired in Δ*tps1* strains during growth under the conditions tested. This conclusion is consistent with work in *A. nidulans* that showed CCR inactivation and constitutive carbon derepression in a *creA^d^* mutant strain did not impair glucose uptake [3].

We next asked whether impaired growth of Δ*tps1* strains on glucose media was due to defects in G6P assimilation into the Δ*tps1* metabolome, such as suggested by the observed G6P accumulation in Δ*tps1* strains. Glucose assimilating defects could result from the misregulation of CCR in these strains, where genes for metabolizing alternative carbon sources are expressed in the presence of glucose. To determine what affect CCR misregulation might have on glucose metabolism in the cell, we undertook a comparative proteomics study of Δ*tps1* and Guy11 mycelial samples (Table S2) to identify at least some of the metabolic processes altered in Δ*tps1*. It should be noted that in this proteomics study, absence of a protein from a sample indicates its level of abundance did not reach the threshold of detection by the current LC/MS/MS set-up used and does not necessarily imply it was not present at all. In support our transcriptional data, proteomic analysis of Δ*tps1* and Guy11 mycelial samples grown in glucose-minimal media showed a putative hexose transporter, MGG_08617 (highlighted in Table S2), was more abundant in Guy11 samples compared to Δ*tps1* samples and is consistent with the role for Tps1 in regulating glucose uptake and metabolism. In addition, malate dehydrogenase (MGG_09872) was detected in Δ*tps1* samples but not the Guy11 proteome (highlighted in Table S2). MGG_09872 was predicted by PSORTII to be localized to the cytoplasm (60.9% probability it is localized to the cytoplasm and 8% it is localized to the mitochondrion), indicating it could be involved in the conversion of malate into oxaloacetate during gluconeogenesis. On the other hand, the enzyme enolase (MGG_10607, involved in glycolysis and gluconeogenesis) and 2,3-bisphosphoglycerate-independent phosphoglycerate mutase (MGG_00901) were not detected in Δ*tps1* samples, but were identified in Guy11 samples, following growth on glucose-containing minimal media (highlighted in Table S2). On the basis of the protein abundance data, some enzymes of gluconeogenesis and glycolysis could be misregulated in Δ*tps1* strains in the presence of glucose compared to Guy11.

**Figure 7:**
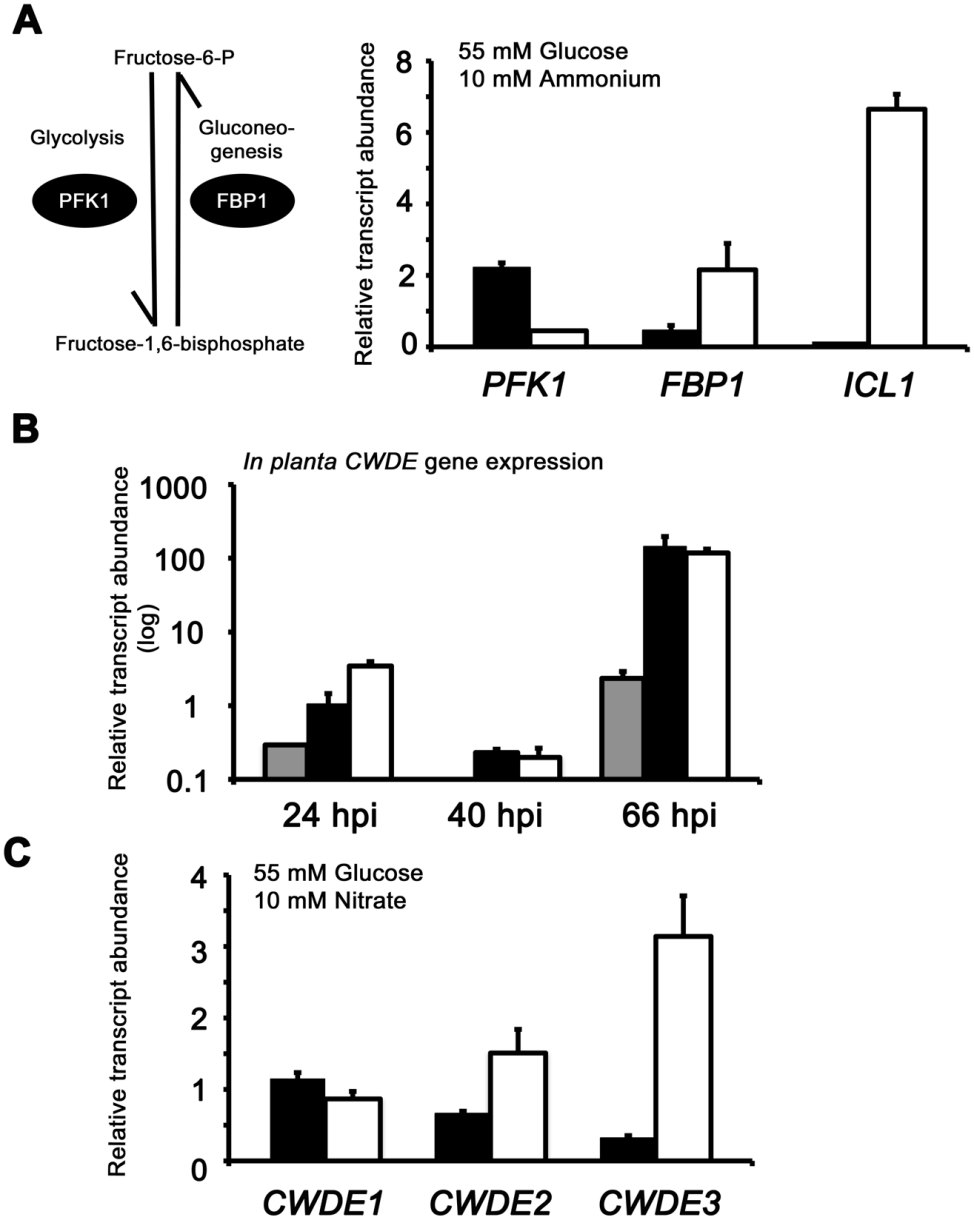
Inactivating CCR in Δ*tps1* strains results in the misregulated expression of genes for assimilating glucose and metabolizing alternative carbon sources. (A) Phosphofructokinase (PFK1) and fructose-1,6-bisphosphatase (FBP1) are glycolytic and gluconeogenic enzymes, respectively, which catalyze the interconversion of fructose-6-phosphate and fructose-1,6-bisphosphate (left panel). Right panel, *PFK1* gene expression is elevated in Guy11 strains (black bars) compared to Δ*tps1* strains (open bars) when grown on minimal media with glucose as sole carbon source. *FBP1* and *ICL1* - encoding isocitrate lyase involved in gluconeogenesis - are elevated in expression in Δ*tps1* strains compared to Guy11 strains when grown on glucose minimal media. Strains were grown in CM media for 48 hr before switching to 55 mM glucose+10 mM NH_4_^+^ minimal media for 16 hr (following [23]). Gene expression results were normalized against expression of the ß-tubulin gene (*TUB2*). Results are the average of at least three independent replicates, and error bars are the standard deviation. (B) Cell wall degrading enzymes (CWDEs) have been shown to be under the control of CCR in *M. oryzae*. To confirm the expression of genes encoding ß-glucosidase 1 (gray bar), feruloyl esterase B (closed bar) and exoglucanase (open bar) during infection by Guy11, their expression was monitored at 24, 40 and 66 hpi and shown to be highly expressed when necrotic lesions were developing. Due to cross-reactivity between fungal and rice ß-tubulin orthologues, gene expression results were normalized against expression of the *M. oryzae* actin gene (*ACT1*). Results are the average of at least three independent replicates, and error bars are the standard deviation. (C) To determine if ß-glucosidase 1, feruloyl esterase B and exoglucanase encoding genes (labeled *CWDE1*, *CWDE2 and CWDE3*, respectively) are subject to Tps1-dependent CCR, their expression was monitored in Guy11 and Δ*tps1* strains following growth in CM for 48 hr and a shift into minimal media with 55 mM glucose and 10 mM NO_3_^−^ for 16 hr. Gene expression results were normalized against expression of the ß-tubulin gene (*TUB2*). Results are the average of at least three independent replicates, and error bars are the standard deviation.

Using the proteomics data as a clue, we sought to determine if Δ*tps1* strains were impaired for glucose assimilation due to the misregulation of genes associated with gluconeogenesis or glycolysis. We studied the expression of *PFK1*, encoding phosphofructokinase and considered the most important control element in the glycolytic pathway due to the irreversible phosphorylation of fructose-6-phosphate to give fructose-1,6-bisphosphate; and *FBP1* encoding fructose-1,6-bisphosphatase I that performs the reverse reaction to PFK1 in gluconeogenesis by dephosphorylating fructose-1,6-bisphosphate to give fructose-6-phosphate. Figure 7A shows that *PFK1* gene expression was elevated in Guy11 strains compared to Δ*tps1* strains on glucose- minimal media. Conversely, *FBP1* was expressed most highly in Δ*tps1* strains on glucose-minimal media. These results are consistant with a previous study which showed phosphofructokinase activity was decreased, and fructose-1,6-bisphosphate activity was increased, in an *Aspergillus* strain carrying an extreme *creA^d^*mutation, compared to wild type, during growth on glucose [33]. We also looked at the expression of a second important gluconeogenic gene, *ICL1*, encoding isocitrate lyase. Isocitrate lyase is necessary for the cleavage of isocitrate to succinate and glyoxylate in the glyoxylate cycle and is required for synthesizing glucose via gluconeogenesis from acetyl-CoA. Isocitrate lyase has also been shown to be subject to CCR control in the tomato pathogen *Fusarium oxysporum* [25]. Figure 7A shows *ICL1* gene expression was significantly elevated in Δ*tps1* strains during growth on glucose compared to Guy11. Therefore, consistent with other CCR mutants, Δ*tps1* strains are upregulated for the expression of genes for alternative carbon source assimilation and down-regulated, relative to Guy11, for the expression of a central gene of glycolysis, indicating poor growth of Δ*tps1* strains on glucose could result from impaired glucose assimilation. It should be noted that *PFK1* is still expressed in Δ*tps1* strains, thus allowing some growth on glucose.

The proteomic data in Table S2 also revealed additional genes likely controlled by Tps1 via CCR. Genes encoding cell wall degrading enzymes (CWDEs) have previously been shown to be glucose-repressed and elevated in expression under glucose-derepressing conditions in *M. oryzae*, although the genes involved in regulating CWDE gene expression in response to carbon source was not previously known [34]. We identified proteins corresponding to putative CWDEs that were more abundantly present in Δ*tps1* samples than Guy11 samples following growth on glucose-containing minimal media (highlighted in Table S2). These included glucan 1,3-beta-glucosidase (MGG_00263, 26-fold more abundant in Δ*tps1* samples than Guy11 samples); a putative cutinase G-box binding protein; chitinase 18-11; feruloyl esterase B; ß-glucosidase 1 and exoglucanase 1 (MGG_00501, MGG_06594, MGG_05529, MGG_09272, and MGG_10712 respectively, detected in Δ*tps1* samples but not detected in Guy11 samples); and D-galacturonic acid reductase (MGG_07463, elevated in abundance in Δ*tps1* samples compared to Guy11). These observations suggested the expression of CWDE-encoding genes were perturbed in Δ*tps1* strains and is consistent with a role for Tps1 in repressing the expression of genes required for metabolizing alternative carbon sources, such as cell wall polysaccharides, in the presence of glucose. To confirm this, we first analyzed the *in planta* expression of the genes encoding ß-glucosidase 1, feruloyl esterase B and exoglucanase (termed *CWDE1*, *CWDE2* and *CWDE3*, respectively) by isolating RNA from infected leaves at 24 hpi (the time of appressorium penetration), 40 hpi (before necrotic lesions had developed) and 66 hpi (when lesions had formed). Figure 7B shows how each gene is highly expressed during the latter stages of infection. Next, we looked at the expression of these genes in Δ*tps1* and Guy11 strains following growth in glucose-media. Figure 7C shows that at least feruloyl esterase B and exoglucanase- encoding genes are derepressed in Δ*tps1* strains compared to Guy11, suggesting they are subjected to Tps1-dependent CCR in the presence of glucose and are misregulated in Δ*tps1* strains.

Taken together, the plate growth, transcriptional and proteomic data describe an essential role for Tps1 in controlling CCR and allowing the fungus to respond correctly to glucose availability.

### Nmr1-3 inhibitor proteins regulate carbon metabolism downstream of Tps1 and independently of Nut1

**Figure 8:**
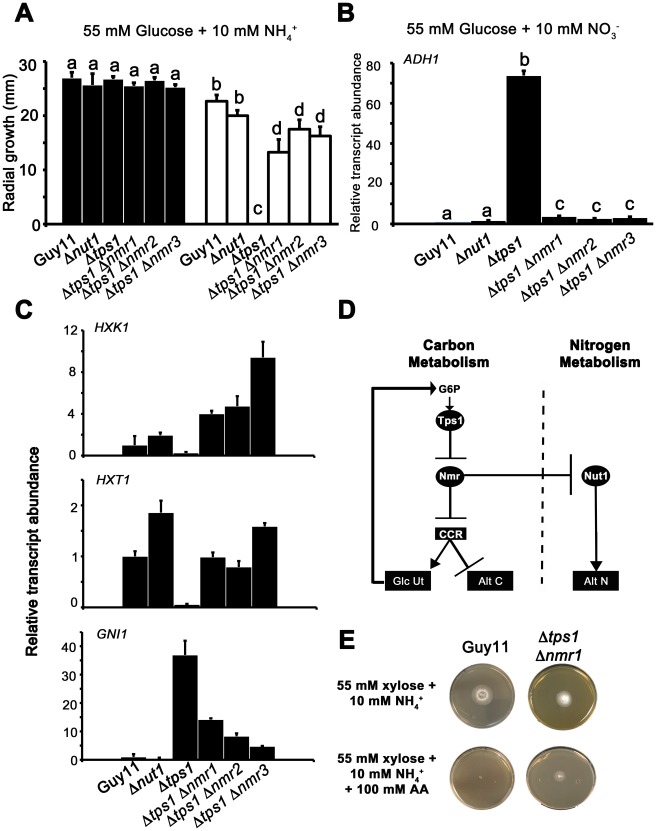
The Nmr1-3 inhibitor proteins regulate CCR independently of Nut1. (A) Guy11, Δ*nut1*, Δ*tps1*, Δ*tps1* Δ*nmr1*, Δ*tps1* Δ*nmr2*, and Δ*tps1* Δ*nmr3* strains were grown on minimal media with 55 mM glucose and 10 mM NH_4_^+^ as sole carbon and nitrogen sources (closed bars), or the same media supplemented with 100 mM of the toxic analogue allyl alcohol (open bars). Strains were grown for 5 days, and radial diameters were measured. Results are the average of three independent replicates. Error bars are standard deviation. Bars with the same letters are not significantly different (*Student's t-test* p≤0.01). (B) The expression of *ADH1* was analyzed in Guy11, Δ*nut1*, Δ*tps1*, Δ*tps1* Δ*nmr1*, Δ*tps1* Δ*nmr2*, and Δ*tps1* Δ*nmr3* strains that were grown in CM media for 48 hr before switching to 55 mM glucose+10 mM NO_3_^−^ minimal media for 16 hr (following [23]). Gene expression results were normalized against expression of the ß-tubulin gene (*TUB2*) and given relative to the expression of *ADH1* in Guy11. Results are the average of at least three independent replicates, and error bars are the standard deviation. (C) The expression of *HXK1* (top panel), *HXT1* (middle panel) and *GNI1* (bottom panel), was analyzed in strains that were grown in CM media for 48 hr before switching to 55 mM glucose+10 mM NO_3_^−^ minimal media for 16 hr. This media was chosen to determine if genes subjected to CCR are expressed independently of Nut1. Gene expression results were normalized against expression of the ß-tubulin gene (*TUB2*) and given relative to the expression of each gene in Guy11. Results are the average of at least three independent replicates, and error bars are the standard deviation. (D) Model for control of CCR and nitrogen metabolite repression in response to G6P. CCR is a signal transduction pathway of unknown components that responds to glucose by inhibiting alternative carbon source utilization (Alt C) and promoting glucose uptake and utilization (Glc Ut) via feed-forward transcriptional regulation. In the absence of glucose, the Nmr1-3 inhibitor proteins inactivate CCR, resulting in carbon derepression, while G6P sensing by Tps1 results in Nmr1-3 inactivation and active CCR. The Nmr1-3 inhibitor proteins also negatively regulate Nut1 to control alternative nitrogen source utilization (Alt N), but Nut1 plays no role in CCR, demonstrating for the first time independent roles for the Nmr1-3 inhibitor proteins in regulating carbon and nitrogen metabolism in response to glucose. (E) Guy11 strains are susceptible to allyl alcohol (AA) toxicity when grown on a derepressing carbon source such as xylose. Consistent with a role for Nmr inhibitor proteins in suppressing CCR, the Δ*tps1* Δ*nmr1* double mutant strain is shown to be partially resistant to 100 AA under carbon derepressing growth conditions (minimal media with 55 mM xylose as sole carbon source), suggesting CCR is at least partially active and suppressing alternative carbon utilization pathways (Alt C), in the absence of glucose, in strains lacking at least Nmr1 activity.

A previous study showed that in response to G6P sensing, Tps1 alleviates Nmr1-3 protein inhibition via modulation of NADPH levels resulting, *inter alia*, in nitrogen derepression [23]. Yeast two-hybrid studies demonstrated Nmr1-3 physically interacted with Asd4, an essential regulator of appresorium formation; Nmr2 interacted with the white collar-2 homologue Pas1; and Nmr1 and Nmr3 interacted with Nut1. Interestingly, deletion of all three *NMR* orthologues was required for full derepression of Nut1 activity under repressing conditions, implying that although not detected in Nut1 binding studies, Nmr2 did have a role in regulating Nut1 activity. In addition, deletion of any one *NMR* gene in the Δ*tps1* background partially restored fungal virulence to Δ*tps1* strains, albeit with reduced lesion sizes compared to Guy11 (shown for Δ*tps1* Δ*nmr1* leaf infection in Figure S5). Thus Δ*tps1* strains have constitutively active Nmr inhibitor proteins, and deleting *NMR* genes in the Δ*tps1* background results in activation of Tps1-dependent gene expression and partial suppression of the Δ*tps1* phenotype [23]. Although Nmr proteins have only previously been described in the literature as mediators of nitrogen metabolism (reviewed in [8]), we sought to establish if Tps1-dependent CCR occurred via Nmr1-3 inhibition in order to shed more light on the role(s) and interaction(s) of Nmr1-3 during infection. We first compared the susceptibility of Δ*tps1*, Δ*tps1* Δ*nmr1*, Δ*tps1* Δ*nmr2* and Δ*tps1* Δ*nmr3* strains to 100 mM AA in glucose minimal media under nitrogen repressing conditions. Δ*nut1* strains were included to determine if the global nitrogen regulator had any influence on AA metabolism. Figure 8A shows that Δ*tps1* strains were susceptible to 100 mM AA in minimal media containing 55 mM glucose and 10 mM NH_4_^+^, whereas the Δ*tps1* Δ*nmr1-3* double mutant strains, like Guy11 and Δ*nut1* strains, were resistant to 100 mM AA and thus restored for CCR. Because Δ*nut1* and Δ*tps1* strains do not grow on plates of nitrate-media, we also looked at the expression of *ADH1* in these strains after growth on CM followed by a switch to nitrate minimal media. Figure 8B shows *ADH1* gene expression was reduced almost 25-fold in Δ*tps1 Δnmr1-3* double mutant strains compared to the Δ*tps1* parental strain and confirms CCR is restored to Δ*tps1* Δ*nmr1-3* double mutant strains relative to Δ*tps1*. Figure 8A and 8B together show that this modulation of CCR by the Nmr inhibitor proteins occurs irrespective of nitrogen source or an active Nut1 protein.

To further explore a role for the Nmr inhibitor proteins in carbon metabolism and CCR, we next looked at the expression of the Tps1-dependent hexose kinase genes, *HXK1* (Figure 8C), *HXK2* (Figure S6A) and *GLK1* (Figure S6B) in Δ*tps1*, the Δ*tps1* Δ*nmr1-3* double mutant strains, and Δ*nut1* compared to Guy11 following growth on minimal media with nitrate. Nitrate was chosen to determine if expression of these genes requires an active Nut1. Hexose kinase gene expression was shown to be elevated in Δ*tps1* Δ*nmr1-3* double mutant strains compared to the Δ*tps1* single mutant strains (Figure 8C and Figure S6A and S6B). Similarly, expression of the putative hexose transporter gene *HXT1* (Figure 8C) was elevated in Δ*tps1* Δ*nmr1-3* double mutant strains compared to Δ*tps1* and in all cases expression was not affected in Δ*nut1* strains compared to Guy11. In addition, the expression of *G6PDH* was shown previously to be reduced in Δ*tps1* strains compared to Guy11 but was restored to wild type levels of expression in the Δ*tps1* Δ*nmr1-3* double mutant strains [23], and Figure S6C shows *G6PDH* gene expression is also independent of Δ*nut1*. Finally, the expression of *GNI1*, subjected to Tps1-dependent CCR, was partially repressed in Δ*tps1* Δ*nmr1-3* double mutant strains compared to Δ*tps1*. Thus, glucose-utilizing genes are expressed, and alternative carbon source utilization is repressed, in Δ*tps1* Δ*nmr1-3* double mutant strains compared to Δ*tps1* strains, in the presence of glucose, while Nut1 is shown to have no role in CCR. Consequently, this is the first description of a role for an NmrA-family protein in regulating both carbon and nitrogen metabolism in a filamentous fungus.

Together, this data suggests the model in Figure 8D, whereby carbon metabolism is regulated by the Nmr1-3 inhibitor proteins independently of Nut1 and in response to G6P sensing by Tps1. Under glucose-repressing conditions, modulation of NADPH levels by Tps1 would inactivate the Nmr inhibitor proteins and result in CCR. Under carbon derepressing conditions, the Nmr inhibitor proteins would be active and suppress CCR. Consistent with this model, we found that Δ*tps1* Δ*nmr1* (but not Δ*tps1* Δ*nmr2* or Δ*tps1* Δ*nmr3*) were able to grow on 100 mM AA in the presence of the derepressing carbon source xylose (Figure 8E), suggesting CCR was at least partially active under derepressing conditions in this strain.

### Nmr1-3 controls Nut1 in response to glucose

**Figure 9:**
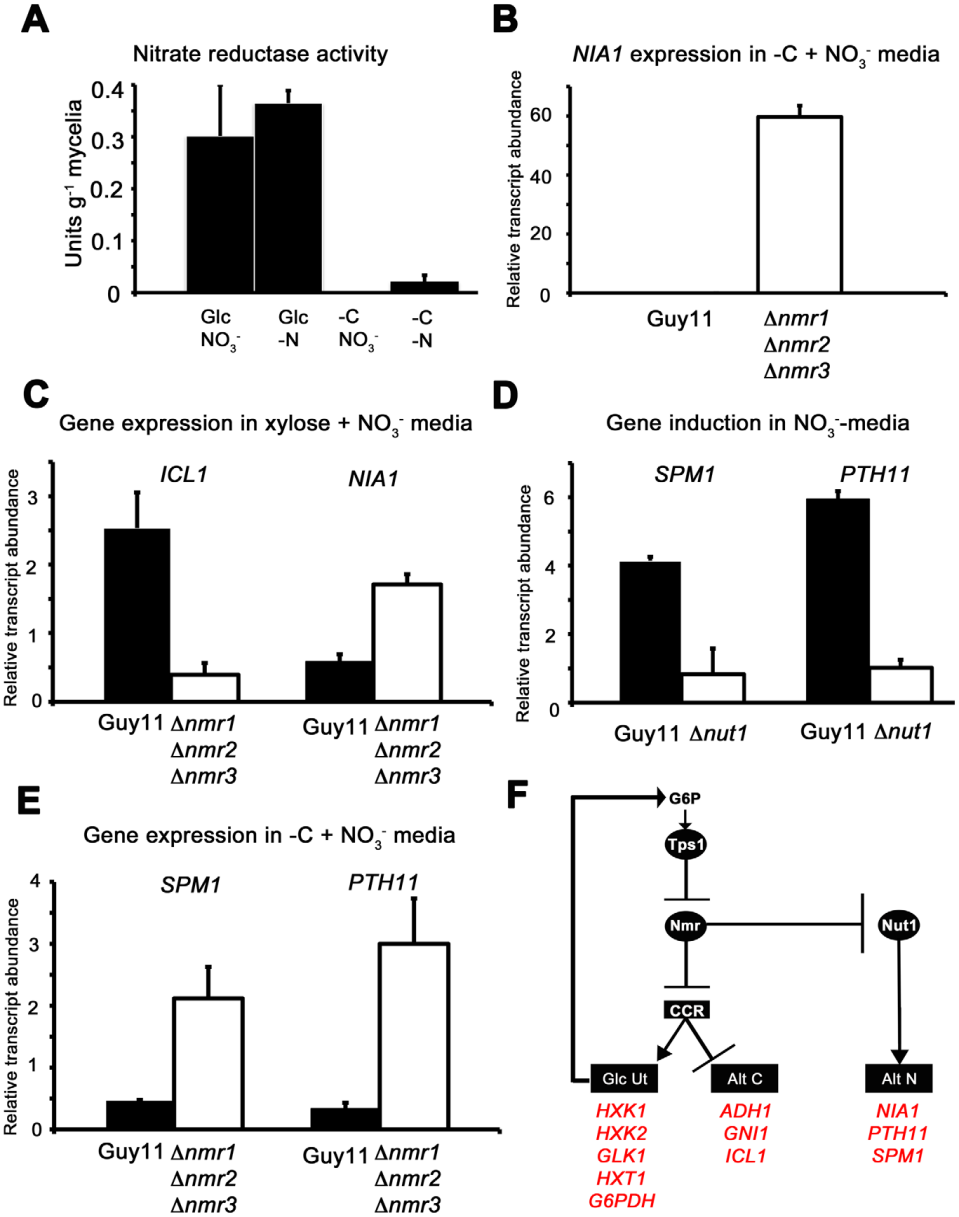
The Nmr1-3 inhibitor proteins regulate nitrogen and carbon metabolism in response to G6P. (A) As predicted by our model in Figure 8D, Nitrate reductase activity is dependent on glucose availability. Nitrate reductase activity was determined as described in [21], where strains were grown in CM media for 48 hr before switching to minimal media containing either 55 mM glucose (Glc) or no carbon source (-C), with 10 mM nitrate (NO_3_^−^) or no nitrogen source (-N). Enzyme activity is given as units of nitrate reductase activity per gram of lyophilized mycelia. Results are the average of at least three independent replicates and bars are standard deviation. (B) *NIA1* expression was analyzed in Guy11 and Δ*nmr1* Δ*nmr2* Δ*nmr3* triple mutant strains following growth in CM for 48 hr followed by growth in nitrate minimal media lacking a carbon source for 16 hr. Gene expression results were normalized against expression of the ß-tubulin gene (*TUB2*). Results are the average of at least three independent replicates, and error bars are the standard deviation. (C) *ICL1* and *NIA1* gene expression was analyzed in Guy11 and Δ*nmr1* Δ*nmr2* Δ*nmr3* triple mutant strains following growth in CM for 48 hr followed by growth in carbon and nitrogen derepressing minimal media (55 mM xylose+10 mM NO_3_^−^). Gene expression results were normalized against expression of the ß-tubulin gene (*TUB2*). Results are the average of at least three independent replicates, and error bars are the standard deviation. (D) To explore how the expression of characterized virulence factors, known to be expressed in nitrogen starvation conditions are controlled, we first confirmed that *SPM1* and *PTH11* are elevated in expression on 55 mM glucose+10 mM NO_3_^−^ minimal media compared to growth on 55 mM glucose+10 mM NH_4_^+^, and that this induction is abolished in Δ*nut1* strains. Gene expression results were normalized against expression of the ß-tubulin gene (*TUB2*) and are relative to their expression in NH_4_^+^-containing minimal media. Results are the average of at least three independent replicates, and error bars are the standard deviation. (E) Having confirmed that *SPM1* and *PTH11* gene expression is nitrate inducible in a Nut1-dependent manner, we next analyzed whether they were regulated by the Nmr1-3 inhibitor proteins. We looked at the expression of these genes on −C+10 mM NO_3_^−^ minimal media in Guy11 and the Δ*nmr1* Δ*nmr2* Δ*nmr3* triple mutant strains, and found they were significantly elevated in expression in the latter strain compared to Guy11. Gene expression results were normalized against expression of the ß-tubulin gene (*TUB2*). Results are the average of at least three independent replicates, and error bars are the standard deviation. (F) Summary of gene regulation discussed in Figure 8 and 9.

With regards to nitrogen metabolism, the model in Figure 8D also predicts that Nmr1-3 should control Nut1 in response to glucose availability and is consistent with our observations that loss of G6P sensing in Δ*tps1* strains locks Nut1 in its inactive form regardless of nitrogen source [21], [23]. Additional evidence for the model proposed in Figure 8D comes from studying the activity of Nut1-dependent processes under different nutritional conditions. Nut1 is required to express *NIA1*, encoding nitrate reductase (NR), under nitrogen derepressing conditions. Nitrate reductase activity was detected in *M. oryzae* mycelial samples grown under NR inducing conditions (glucose and NO_3_^−^ minimal media, Figure 9A) but was absent following growth under nitrogen repressing conditions, ie glucose and NH_4_^+^ [23]. NR activity was also not detected in NR induction media lacking a source of carbon (−C+NO_3_^−^, Figure 9A), consistent with previous observations in *A. nidulans* which showed NR activity rapidly disappeared from mycelial samples switched from NR induction media into media lacking a carbon source [35]. In *M. oryzae*, NR activity was also not detected in mycelia grown under nitrogen and carbon starvation conditions (-C –N, Figure 9A). Interestingly NR activity was detected in our *M. oryzae* mycelia grown in glucose minimal media lacking a nitrogen source (-N, Figure 9A). This is different to the observations by Hynes of *A. nidulans* NR activity [35], where absence of an inducer resulted in rapid loss of NR activity, but consistent with a previous *M. oryzae* report that showed *NIA1* expression was elevated under nitrogen starvation conditions in *M. oryzae* compared to growth on nitrate media in the presence of glucose [36]. Figure S7A confirms that *NIA1* is expressed in the absence of inducer, but not the absence of a carbon source, in wild type Guy11 strains. In addition, *NIA1* is expressed in condia and appressoria in the absence of an inducer [23], and we show in Figure S7B that in appressoria, *NIA1* expression is dependent on Tps1.

In *A. nidulans*, although NR activity requires an inducer, several other activities - such as acetamidase, histidase and formamidase - are present at high levels in nitrogen starvation media in the absence of an inducer. Todd et al [37] examined the expression of *amdS* to demonstrate for *A. nidulans* that under nitrogen starvation conditions, in the presence of glucose, AreA located to the nucleus. AreA nuclear accumulation was rapidly reversed by the addition of an exogenous nitrogen source, and was not seen in nitrogen starvation media lacking a carbon source. Our results might be consistent with this model of AreA/Nut1 activity in *M. oryzae* under at least some starvation conditions where *NIA1* gene expression does not appear to require an inducer.

The model in Figure 8D suggests carbon metabolism and nitrogen metabolism are regulated by the Nmr1-3 inhibitor proteins in response to glucose, and Figure 9A and Figure S7A confirm NR activity and *NIA1* gene expression is abolished in carbon starvation media in the presence of nitrate. However, the model in Figure 8D predicts that inactivating the Nmr1-3 inhibitor proteins should result in *NIA1* gene expression in carbon starvation media. Consistent with this hypothesis, Figure 9B shows that *NIA1* gene expression is significantly elevated in the Δ*nmr1* Δ*nmr2* Δ*nmr3* triple mutant [23] following growth on −C+NO_3_^−^ media compared to Guy11.

The model also predicts that in Guy11, under carbon and nitrogen derepressing conditions (for example 55 mM xylose+10 mM NO_3_^−^), Nmr1-3 inhibitor proteins would be active, resulting in both Nut1 inhibition and CCR repression. The outcome of this growth condition is expected to be both decreased *NIA1* expression and increased expression of genes for alternative carbon source utilization. Conversely, growth of the Δ*nmr1* Δ*nmr2* Δ*nmr3* triple mutant under the same conditions should result in increased *NIA1* gene expression, and active CCR and decreased expression of alternative carbon utilization genes, relative to Guy11 (Figure 8D). Figure 9C shows this to be the case, with *ICL1* gene expression reduced, and *NIA1* gene expression elevated, in Δ*nmr1* Δ*nmr2* Δ*nmr3* triple mutant strains compared to Guy11 following growth on 55 mM xylose and 10 mM NO_3_^−^.

Taken together, these results support a role for Tps1 in integrating carbon and nitrogen metabolism such that in glucose-rich conditions, Tps1 senses G6P and inactivates Nmr1-3 regardless of nitrogen source, resulting in active Nut1 and CCR. Conversely, in the absence of G6P, Nmr1-3 would simultaneously repress CCR and nitrogen metabolism regardless of nitrogen source.

### The role of Nmr1-3 inhibition in the expression of known virulence factors under nitrogen starvation conditions

In *M. oryzae* and other plant pathogens, it has been noted that virulence-associated gene expression is induced on glucose minimal media lacking a nitrogen source [36], [38], and Figure 8D suggests one mechanism by which these genes could be controlled during infection. To explore this further we looked at the expression of two genes essential for virulence and encoding the vacuolar serine protease Spm1 [36] and the plasma membrane protein Pth11 [39]. *PTH11* gene expression had previously been shown to be under Tps1 control [21] and both *PTH11* and *SPM1* were shown to be elevated in expression under nitrogen starvation conditions compared to nitrate inducing conditions [21]. However, whether the expression of *PTH11* and *SPM1* was ammonium-repressible, and whether that repression occured via Nmr1-3 control of Nut1, was not known. Figure 9D shows therefore that in Guy11, both *SPM1* and *PTH11* gene expression is induced in NO_3_^−^ media compared to NH_4_^+^ media (with 55 mM glucose in both cases), and that this induction is dependent on Nut1. Figure 9E shows that *SPM1* and *PTH11* are also regulated by the Nmr1-3 inhibitor proteins in response to glucose whereby expression of both genes is elevated in the Δ*nmr1* Δ*nmr2* Δ*nmr3* strain during growth on carbon starvation media in the presence of nitrate, compared to Guy11. Figure 9F summarizes the transcriptional data in Figure 8 and Figure 9 to show how carbon and nitrogen metabolism is integrated in response to G6P availability, and how this could provide a framework for understanding how known virulence genes, expressed under nitrogen starvation conditions, are regulated during infection.

### An extragenic forward suppressor screen identified *MDT1*, encoding a MATE–family efflux pump, as an additional regulator of the CCR signal transduction pathway in *M. oryzae*

At the start of this study, nothing was known about the downstream target(s) of Tps1 and Nmr1-3 involved in CCR, or what additional factors constitute the CCR signaling pathway in *M. oryzae*. Because *CREA* deletion mutants can often not be obtained [25], we used our Δ*nut1* deletion strain in a forward genetics screen to identify components of CCR by selecting for extragenic suppressors of Δ*nut1* that were restored in their ability to utilize proline or glucosamine in the presence of glucose. Our rationale for defining CCR in *M. oryzae* lies in understanding how carbon metabolism is regulated in *M. oryzae* and how such nutrient adaptability contributes to pathogenicity during plant infection. *Agrobacterium tumefaciens*-mediated mutagenesis using the binary vector pKHt [40] randomly introduced T-DNA into the genome of a Δ*nut1* parental strain, and the resulting suppressor strains were selected for growth on minimal media containing 10 mM glucose with 10 mM proline or 10 mM glucosamine as nitrogen source. We obtained a total of six transformats on 10 mM glucose+10 mM proline (Δ*nut1 Supp 3121021–*Δ*nut1 Supp 3121026*) and one transformant on 10 mM glucose+10 mM glucosamine (Δ*nut1 Supp 312104*). Three transformants selected on 10 mM glucoe+10 mM proline were lost due to *Agrobacterium* contamination before we were able to identify the disrupted gene. For the remaing four strains (Δ*nut1 Supp 312102*, Δ*nut1 Supp 3121023*, Δ*nut1 Supp 3121025* and Δ*nut1 Supp 312104*), inverse PCR and the known T-DNA sequence [40] were used to identify genes that had been disrupted by T-DNA insertion in our suppressor strains. Interestingly, all four Δ*nut1* suppressor strains resulted from T-DNA insertions into the same 3′ coding region of *MDT1* encoding a MATE-family efflux pump [27] (Table S3, Figure S8A). Specifically, all resulted from T-DNA insertions immediately 3′ to nucleotide 1503, except Δ*nut1 Supp 3121023* which resulted from insertion of T-DNA immediately 3′ to nucleotide 1502. The position of these insertions could indicate that all the suppressors generated were not independent transformants. However, Δ*nut1 Supp 312104* was selected on 10 mM glucose+10 mM glucosamine using different Guy11 mycelial samples to the suppressors selected on glucose+proline. In addition, previous reports of using *Agrobacterium* -mediated mutagenesis in *Arabidopsis* [41] and *Magnaporthe* [42] demonstrated nonrandom integration of T-DNA, and “hotspots” of integration were determined. Therefore, our suppressors could result either from multiple insertions of *Agrobacterium* T-DNA into a “hotspot” region within *MDT1*, or result from clones of one transformant isolated during selection. The suppressor strains generated in Table S3, regardless of the selection media used, were able to grow on both glucosamine and proline as nitrogen source compared to the Δ*nut1* parental strain, and we arbitrarily chose Δ*nut1 Supp 3121022* for further characterization. To confirm *MDT1* as the suppressing locus in Δ*nut1* suppressor strains, a Δ*nut1* Δ*mdt1* double deletion strain was generated by homologous gene replacement of *MDT1* in the Δ*nut1* background (Figure S6B). *MDT1* was also deleted in Guy11 to generate a single Δ*mdt1* deletion strain that was subsequently complemented with the full length *MDT1* coding region.

**Figure 10:**
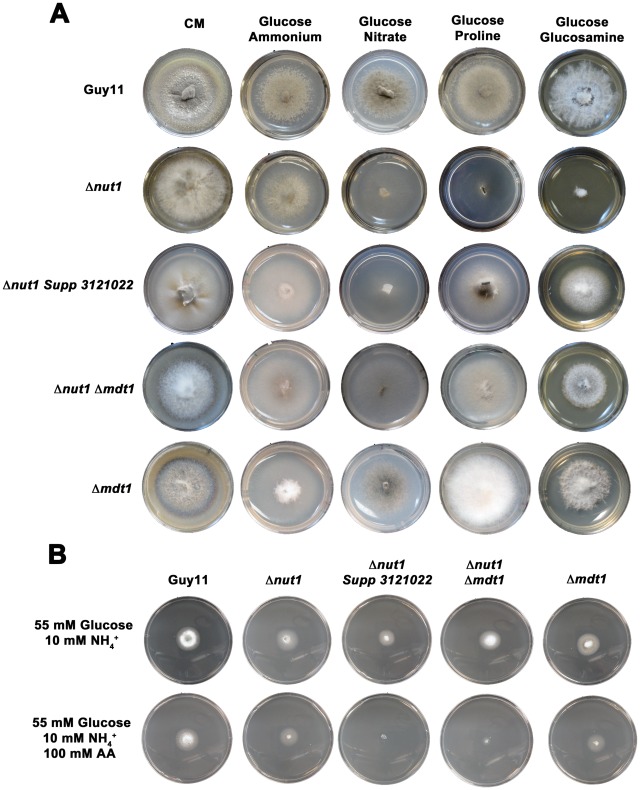
Disruption of Mdt1 function affects carbon metabolism. (A) Strains were grown for 10 days on CM or minimal media supplemented with 10 mM of the appropriate carbon and nitrogen source. Like the Δ*nut1* parental strain, both Δ*nut1 Supp 321022* extragenic suppressor strains and Δ*nut1* Δ*mdt1* double deletion strains were unable to grow on NO_3_^−^ - containing media. Unlike the Δ*nut1* parental strain, both *MDT1* disruption strains were restored for growth on proline and glucosamine as nitrogen source, indicating T-DNA insertion or homologous gene replacement of *MDT1* resulted in carbon derepression in the presence of glucose. (B) Disruption of *MDT1* in the Δ*nut1* background resulted in strains that were carbon catabolite derepressed and significantly reduced in growth on 55 mM glucose+10 mM NH_4_^+^ minimal media with 100 mM AA compared to growth on NH_4_^+^ minimal media alone. Single Δ*mdt1* deletion strains were less sensitive to 100 mM AA on this media.

Figure 10A shows how both the Δ*nut1 Supp 3121022* suppressor strain and the Δ*nut1* Δ*mdt1* double deletion strain, like the Δ*nut1* parental strain, could not use nitrate as a sole nitrogen source. Unlike Δ*nut1* deletion strains, however, Δ*nut1 Supp 3121022* and Δ*nut1* Δ*mdt1* strains could grow on proline and glucosamine as nitrogen source, and were sensitive to 100 mM AA in 55 mM glucose+10 mM NH_4_^+^ minimal media (Figure 10B), indicating they were derepressed for carbon metabolism in the presence of glucose.

The Δ*mdt1* single mutant strains could grow on nitrate, glucosamine and proline as nitrogen sources, and were slightly more sensitive to glucose minimal media with 100 mM AA than Guy11. Δ*mdt1* strains were also reduced in growth on minimal media with 10 mM glucose+10 mM NH_4_^+^ (discussed below).

### *MDT1* encodes a predicted membrane-spanning protein and is expressed during appressoria development

*MDT1* is a member of the Multidrug and Toxin Extrusion (MATE) gene family found in bacteria, archaea and eukaryotes [43], [44], [45]. They have a wide range of cellular substrates and function as fundamental transporters of metabolic and xenobiotic organic cations in kidneys [46]; transporters of organic anions such as citrate in plants [47]; and contribute to antimicrobial drug resistance and protection against ROS damage in bacteria [48]. In *M. oryzae*, the *MDT1* locus, MGG_03123, is one of three loci encoding putative MATE-family efflux pumps, the other two being MGG_04182 and MGG_10534 [49]. MGG_03123 consists of 2510 nucleotides, has three predicted introns, and encodes a 748 amino acid protein. PSortII analysis predicted the gene product has a 73.9% chance of being localized to the plasma membrane, and a 26.1% chance of being localized to the endoplasmic reticulum. TMpred and PSIPRED analysis predicted the protein carries 12 membrane-spanning helices located in the C-terminal region of the protein. An EST corresponding to MGG_03123,10_GI3391884.f, was detected in appressorial stage specific cDNAs deposited at the *M. oryzae* community database (www.mgosdb.org/).

### *MDT1* is required for sporulation and plant infection but not appressorium formation

**Figure 11:**
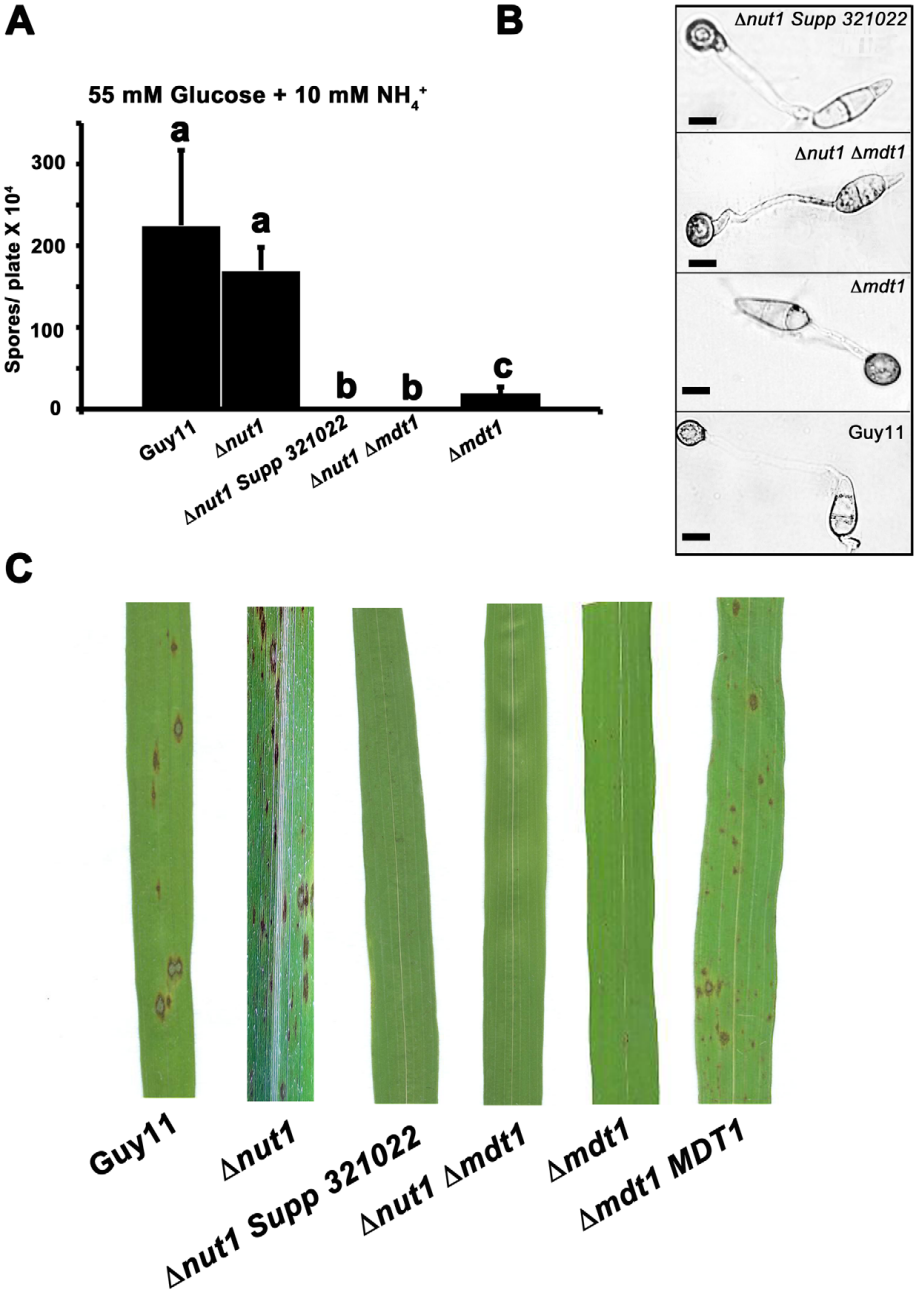
Disrupting Mdt1 function affects sporulation and pathogenesis. (A) *MDT1* disruption mutants were impaired in spore production on minimal media. Spores were harvested from plates following 12 days of growth. Values are the mean of at least three independent replicates. Error bars are standard deviation. Bars with the same letter are not significantly different (*Student's t-test* p≤0.01). (B) The *Δnut1 Supp 321022* suppressor strain, the Δ*nut1* Δ*mdt1* double deletion strain and the Δ*mdt1* single mutant form appressoria on artificial hydrophobic surfaces. Spores of Guy11, the *Δnut1 Supp 321022* suppressor strain, the Δ*nut1* Δ*mdt1* double deletion strain and Δ*mdt1* strains were applied to plastic cover slips. Scale bars are 10 µM. (C) The MATE-family efflux pump Mdt1 is essential for pathogenesis in Δ*nut1* strains. Because of the reduced sporulation rates of *MDT1* disruption strains, spores were inoculated onto rice leaves at a low rate of 2×10^4^ spores/mL. Compared to Guy11 and Δ*nut1* parental strains, Δ*mdt1*, Δ*nut1* Δ*mdt1* and Δ*nut1 Supp 321022* strains were unable to cause the necrotic lesions associated with successful rice infection. Introducing the full length *MDT1* coding region into Δ*mdt1* strains restored pathogenicity in Δ*mdt1 MDT1* complementation strains.

Disruption of *MDT1* by T-DNA insertion or homologous recombination, in wild type or Δ*nut1* backgrounds, resulted in significant reductions in spore production on minimal media with 55 mM glucose and 10 mM NH_4_^+^ compared to the Guy11 and Δ*nut1* parental strains (Figure 11A). Sufficient spores were harvested from CM plates to show that after 24 hrs, spores of Δ*mdt1*, Δ*nut1* Δ*mdt1* and Δ*nut1 Supp 321022* strains formed appressorium normally on hydrophobic surfaces compared to Guy11 (Figure 11B). However, despite forming appressoria, Δ*mdt1*, Δ*nut1* Δ*mdt1* and Δ*nut1 Supp 321022* strains were unable to establish disease when inoculated onto rice leaves (Figure 11C). To ensure loss of pathogenicity was solely due to the loss of a functional *MDT1* gene, we show Δ*mdt1* strains complemented with the full length *MDT1* gene are restored for pathogenicity (Figure 11C). Thus *MDT1* is not required for appressorium development but is essential for both full sporulation and rice blast disease and is a new determinant of virulence in *M. oryzae*.

### *MDT1* is involved in citrate efflux and carbon regulation

We sought to identify the likely function of Mdt1 in order to understand how a MATE-family efflux pump might regulate carbon metabolism and mediate the fungal-host plant interaction. Only one other MATE-family transporter had previously been described in fungi, Erc1 from *S. cerevisiae*, which functions to confer fungal resistance to the toxic methionine analog ethionine [50]. We observed that although Guy11 is sensitive to the addition of 50 mM ethionine to glucose minimal media, *MDT1* disruption strains did not demonstrate increased susceptibility compared to Guy11, suggesting Mdt1 is not involved in ethionine efflux in *M. oryzae* (Figure S9A).

Considering *Δnut1 Supp3121022* and Δ*nut1* Δ*mdt1* strains were carbon derepressed in the presence of glucose (Figure 10B), we sought to determine if Mdt1 might be involved in glucose uptake and phosphorylation in the cell. As noted previously, a class of carbon derepressed mutants of *A. nidulans* were found to result from defective glucose uptake [3] and were consequently resistant to both the toxic glucose analogue 2-deoxyglucose (2-DOG), and the toxic sugar sorbose [32] during growth under carbon derepressing conditions. When grown on carbon derepressing minimal media comprising 10 mM xylose and 10 mM NH_4_^+^ as sole carbon and nitrogen sources, we observed, however, that disruption of *MDT1* in all backgrounds tested (wild type and Δ*nut1*) did not confer resistance to 50 µg/mL 2-DOG or 5 mM sorbose in *M. oryzae* (Figure S9B). This suggests Mdt1 is not involved in glucose uptake in *M. oryzae*.

In bacteria, the MATE-family efflux protein NorM protects GO-deficient strains against the deleterious effects of exogenous reactive oxygen species (ROS) [48]. As *M. oryzae* transitions from the surface of the leaf to the underlying tissue, it encounters basal plant defense strategies in the form of a plant-derived oxidative burst, which the fungus needs to neutralize in order to establish infection [51]. We wondered if Mdt1 might play a similar role to NorM in protecting *M. oryzae* against ROS, and if loss of this protection in *MDT1* disruption strains might result in the observed loss of pathogenicity. However, Figure S9C shows how Δ*nut1* Δ*mdt1* double mutant and Δ*mdt1* single mutant strains were not significantly more sensitive to oxidative stress than wild type, as evidenced by their ability to grow like wild type and Δ*nut1* parental strains on CM supplemented with 10 mM H_2_O_2_. In contrast, Des1 is an *M. oryzae* gene product necessary for neutralizing plant ROS during infection [51]. Δ*des1* mutant strains are unable to detoxify plant ROS and are severely attenuated in growth on CM containing only 3 mM H_2_O_2_ compared to wild type. Therefore Figure S9C suggests that, compared to Des1, the role of Mdt1 in protecting the fungus against ROS during infection is very minor.

Considering the wide range of MATE substrates demonstrated in other organisms, additional roles for Mdt1 could also include conferring toxin resistance during growth *in planta* through the extrusion of plant-derived defense compounds from the fungal cell, such as has been reported previously in *M. oryzae* for the transmembrane ATP binding cassette (ABC) proteins, Abc1 [52] and Abc3 [53]. However, it should be noted that unlike Δ*abc1* and Δ*abc3* mutant strains, *MDT1* disruption strains evince physiological defects and inactivation of CCR under glucose-rich conditions in the absence of the plant host. This suggests Mdt1 has a major physiological role in carbon metabolism and any additional role(s) it might have in mediating resistance to plant toxins during infection is likely to be a minor function of this efflux pump.

**Figure 12:**
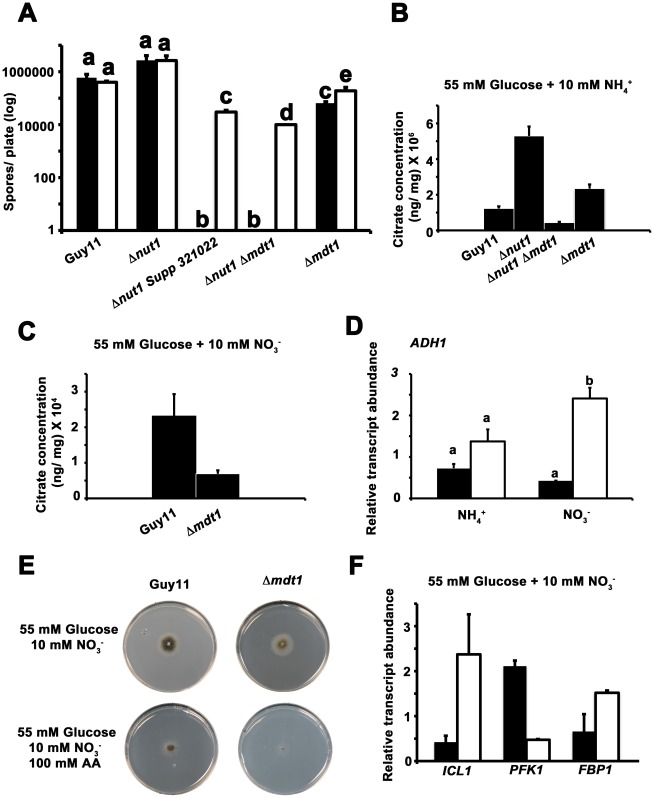
Elucidating the function of the Mdt1 efflux protein. (A) Sporulation of the Δ*nut1 Supp 321022* suppressor strain, the Δ*nut1* Δ*mdt1* double deletion strain, and the Δ*mdt1* single deletion strain, but not the Guy11 or Δ*nut1* parental strains, was significantly increased (*Student's t-test* p≤0.05) on minimal media comprising 55 mM glucose and 10 mM NH_4_^+^ and containing ten-fold more zinc (open bars) than the same media with standard zinc concentrations (closed bars). Spores were harvested from plates following 12 days of growth. Log scale is used. Values are the average of three independent replicates and error bars are the standard deviation. (B) Strains were grown in CM for 48 hr before switching to minimal media with 55 mM glucose and 10 mM NH_4_^+^ or (C) 10 mM NO_3_^−^ as sole nitrogen sources for 16 hr. Citrate was measured in the media using LC-MS/MS and quantified against known concentrations of citrate. Values are the mean of at least three independent replicates. Error bars are standard deviation. (D) *ADH1* gene expression was analyzed in strains of Guy11 (closed bars) and Δ*mdt1* (open bars) after 16 hr expression in minimal media with 10 mM NO_3_^−^ or 10 mM NH_4_^+^ as sole nitrogen source, and 55 mM glucose as carbon source, following a switch from CM media. Gene expression results were normalized against expression of the ß-tubulin gene (*TUB2*). Results are the average of at least three independent replicates, and error bars are the standard deviation. Bars with the same letter are not significantly different (*Student's t-test* p≤0.05). (E) Δ*mdt1* strains are carbon derepressed on minimal media with 55 mM glucose+10 mM NO_3_^−^ and show increased sensitivity to 100 mM AA on this media compared to Guy11. Strains were grown for 5 days on 85 mm plates. (F) Compared to Guy11 strains, growth of Δ*mdt1* strains on minimal media with 55 mM glucose+10 mM NO_3_^−^ results in changes to *ICL1*, *PFK1* and *FBP1* gene expression. Gene expression results were normalized against expression of the ß-tubulin gene (*TUB2*). Results are the average of at least three independent replicates and error bars are the standard deviation.

In *Arabidopsis thaliana* and rice (*Oryza sativa*), root-associated MATE-family transporters, AtFRD3 and OsFRDL1 respectively, are indirectly involved in cellular metal uptake and homeostasis [54]–[57]. MATE proteins likely do not transport metal ions directly but are proposed to secrete citrate that chelates extracellular metal ions and conditions their translocation into the cell by other systems [47], [56], [58]. MATE proteins involved in citrate efflux have also been described for sorghum [59], barley [60], maize [61] and wheat [47]. In addition, fungi have been shown to secrete citrate, where it is considered overflow metabolism similar to that seen during growth on excess glucose [62]. We first sought to determine if Mdt1 was involved in metal uptake in *M. oryzae*. Figure 12A demonstrates that compared to growth on minimal media containing 55 mM glucose and 10 mM NH_4_^+^, growth on the same media supplemented with ten-fold the normal concentration of zinc, but not copper or iron (not shown), significantly increased sporulation rates in Δ*nut1 Supp 312022*, Δ*nut1* Δ*mdt1* double deletion and Δ*mdt1* single deletion strains, but not Guy11 or Δ*nut1* parental strains, following 12 days of growth. This suggests the *MDT1* disruption strains were impaired in zinc uptake.

Next, to explore the role of zinc metabolism during infection, we generated a deletion mutant of *ZAP1* (MGG_04456) in *M. oryzae* by homologous gene replacement. Zap1 is a transcription factor described in *Saccharomyces cerevisiae* that regulates the expression of genes encoding zinc uptake systems [63]. *S. cerevisiae* strains carrying mutations in this gene grow poorly on zinc-depleted media. To test whether *MoZAP1* is an *Sczap1* functional homologue that regulates zinc homeostasis and acquisition, we measured the sporulation rates of Δ*zap1*-carrying strains of *M. oryzae* grown on minimal media with our standard concentration of zinc (1×Zn) and on minimal media with a 100-fold reduction in zinc (1∶100×Zn) (Figure S10A). Compared to Guy11 on the same media, sporulation of Δ*zap1* strains was significantly reduced on 100-fold reduced zinc media compared to standard media, thus demonstrating these mutant strains were likely impaired for zinc acquisition on zinc-depleted media. When applied to rice leaves, Δ*zap1* strains were determined to be fully pathogenic (Figure S10B), indicating the physiological effects of growth under zinc-limiting conditions demonstrated for Δ*zap1* is not deterimental to pathogenicity. However, because complete loss of growth and sporulation of Δ*zap1* strains on zinc-limiting media was not observed, other zinc acquisition systems must be operational in these strains, and their elucidation warrants further investigation.

Metal homeostasis in plant roots is dependent on MATE proteins that, in some cases, have been shown to extrude citrate. In addition, fungi excrete citrate during growth under excess glucose conditions [62]. To determine if Mdt1 was involved in citrate efflux, we grew *MDT1* deletion strains and parental strains in CM for 48 hr then switched the mycelia to ammonium minimal media for 16 hr (Figure 12B). Δ*nut1 Supp 321022* was omitted from these studies due to uncertainty about the effects any additional unidentified T-DNA insertions might have on citrate efflux. After 16 hr, the filtrate was harvested and citrate exudate was quantified using LC-MS/MS. Under these conditions, media of the single Δ*mdt1* deletion mutant did not contain less citrate than Guy11. However, media of Δ*nut1* contained significantly more citrate than Guy11, while media of the Δ*nut1* Δ*mdt1* double mutant was reduced in citrate compared to both parental strains. Therefore on ammonium media, loss of Mdt1 leads to reduced citrate efflux in the Δ*nut1* background.

On nitrate minimal media, the Δ*mdt1* deletion mutant was shown to produce less citrate in the media than Guy11 (Figure 12C) suggesting it might therefore be necessary for citrate efflux under these growth conditions. Δ*nut1* and Δ*nut1* Δ*mdt1* were not analyzed in this media because they are essentially nitrogen starved on nitrate and might generate spurious results regarding citrate efflux. Interestingly, reduced Mdt1-dependent citrate efflux appeared to correlate with carbon derpression such that in Δ*mdt1* strains, *ADH1* gene expression was not different to Guy11 during growth on minimal media with glucose and ammonium, but was significantly elevated compared to Guy11 on minimal media with glucose and nitrate (Figure 12D). Indeed, Figure 12E shows that on minimal media with 55 mM glucose and 10 mM NO_3_^−^, Δ*mdt1* strains were carbon derepressed in the presence of glucose and highly sensitive to 100 mM AA compared to Guy11. To determine if loss of Mdt1 function and reduced citrate efflux correlated with changes in other carbon metabolic processes, we looked at the expression of *ICL1*, *PFK1* and *FBP1* in Guy11 and Δ*mdt1* strains following growth on minimal media with 55 mM glucose and 10 mM NO_3_^−^ (Figure 12F). Under these conditions (and similar to Δ*tps1* strains), Δ*mdt1* expressed genes for alternative carbon source metabolism (*ICL1*) and gluconeogenesis (*FBP1*) more highly than Guy11 but was reduced in the expression of the glycolytic gene *PFK1*. Therefore, extrusion of citrate by Mdt1 is context-dependent (ie dependent on growth nutrient conditions and genetic background) and is likely required during overflow metabolism to remove excess citrate from the cell, while loss of Mdt1 function in the same conditions results in CCR activation in the presence of glucose.

Taken together, we propose the major physiological role for Mdt1 during infection and growth is in mediating carbon metabolism via extrusion of citrate, thus contributing to *in planta* nutrient adaptation.

### *MDT1* is hypostatic to *TPS1*

**Figure 13:**
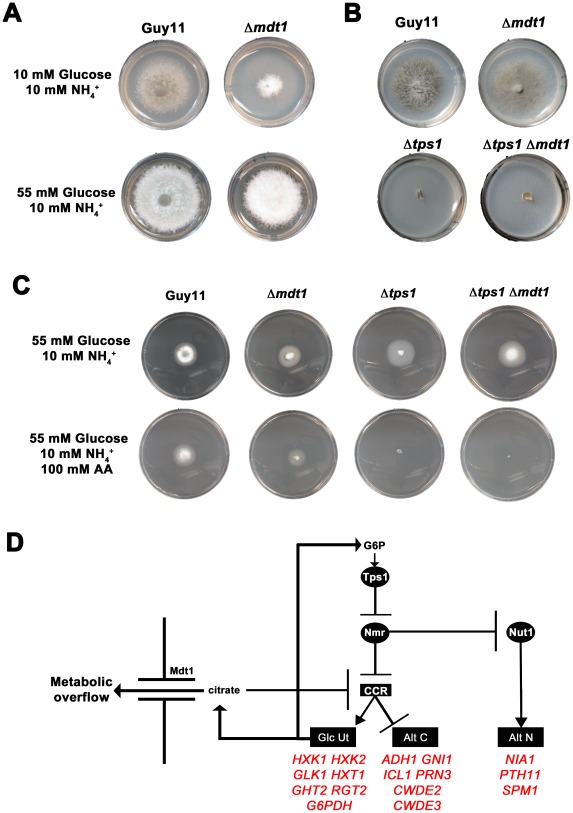
*TPS1* is epistatic to *MDT1* in the regulation of CCR. (A) Like Δ*tps1* strains, Δ*mdt1* strains are impaired for growth on NH_4_^+^-minimal media with 10 mM glucose compared to Guy11, but grow stronger on NH_4_^+^-minimal media with 55 mM glucose. (B) Unlike Δ*tps1* and Δ*tps1* Δ*mdt1* strains, single Δ*mdt1* deletion strains can utilize 10 mM NO_3_^−^ as a nitrogen source suggesting *TPS1* is epistatic to *MDT1*. (C) Δ*mdt1* single mutant strains are less sensitive to 55 mM Glc+10 mM NH_4_^+^ minimal media containing mM AA than Δ*tps1* and Δ*tps1* Δ*mdt1* strains. Strains were grown for 5 days on 85 mm plates. (D) We propose under sugar-rich conditions, such as those found in the interior of the rice leaf, G6P sensing by Tps1 inactivates the Nmr1-3 inhibitor protiens via elevated NADPH levels as described in [23]. This results in both the derepression of GATA factor activity and the activation of CCR. CCR inhibits the expression of genes for alternative carbon source utilization and promotes the expression of genes for glucose utilization. Excess glucose would lead to overflow metabolism and citrate accumulation in the cell, and Mdt1 is necessary for the extrusion of citrate. Because loss of Mdt1 function inactivates CCR, we propose that citrate accumulation in the cell directly or indirectly inhibits CCR downstream of Tps1 and the Nmr1-3 inhibitor proteins. When G6P is exhausted, such as when the fungus has killed the leaf cell and no more photosynthesis is occurring, the Nmr1-3 inhibitor proteins would become active, blocking CCR and promoting the expression of genes for alternative carbon source utilization, including the large number of CWDEs first reported here. Glc is glucose. Glc Ut is glucose utilization. Alt C is alternative carbon source utilization. GATA represents the Asd4 and Nut1 GATA family transcription factors that are known to form physical interactions with Nmr1 [23].

Δ*mdt1* strains grew poorly on minimal media with 10 mM glucose (Figure 10A), but were not impaired in glucose uptake and phosphorylation (Figure S9B). They were, however, misregulated for genes associated with alternative carbon source utilization and assimilation (Figure 12D and 12F). In addition, Figure 13A demonstrates that growth of Δ*mdt1* was improved on minimal media with 55 mM glucose compared to 10 mM glucose, while growth of Guy11 on either media is not affected. These results are similar to those seen for Δ*tps1*, suggesting Mdt1 also functions to regulate glucose metabolism. To determine the genetic relationship between Tps1 and Mdt1, we constructed a Δ*tps1* Δ*mdt1* double mutant and compared its growth to Δ*mdt1* and Δ*tps1* single deletion strains. Growth of Δ*mdt1*, but not Δ*tps1* Δ*mdt1* or Δ*tps1* strains, on minimal media with 55 mM glucose and 10 mM NO_3_^−^ (Figure 13B) confirms that Mdt1 regulates CCR downstream of Tps1 and after the Nmr1-3 mediated pathway branch to nitrogen metabolism. In addition, Δ*tps1* Δ*mdt1* double deletion strains, like Δ*tps1*, were highly sensitive to 100 mM AA on minimal media with ammonium compared to Δ*mdt1* single mutant strains, suggesting Tps1- and Mdt1- dependent regulation of CCR can occur in response to different signals, demonstrated in Figure 13C. In this model, in the presence of glucose, CCR is active due to G6P sensing by Tps1 and the inactivation of the Nmr1-3 inhibitor proteins. Under conditions of excess glucose, such as might be found in the photosynthesizing leaf, overflow metabolism results in citrate production, which is extruded from the cell by Mdt1. We propose perturbed citrate efflux from the cell can directly or indirectly inactivate CCR, slowing the uptake and metabolism of glucose and providing a mechanism for reducing overflow metabolism. Finally, as disease progresses and external glucose is exhausted, loss of G6P sensing by Tps1 inactivates CCR to allow the metabolism and assimilation of alternative carbon sources, such as cell wall polysacharides. Thus together, Tps1 and Mdt1 represent sensitive monitors of carbon metabolism that allow the fungus to adapt to fluctuating qualities and quantities of carbon sources during infection.

### Conclusions and significance

Recently, Tps1 was shown in the rice blast fungus to integrate nitrogen metabolism with G6P availability, and we sought to determine what role it might play in regulating carbon utilization during infection. Here we demonstrate that Tps1, via the Nmr1-3 inhibitor proteins, regulates CCR in the presence of G6P to ensure the preferential utilization of glucose over less favourable carbon compounds. This confirms that in filamentous fungi, glucose phosphorylation, rather than signaling by individual hexokinase proteins, is the first step in signaling glucose repression [11]. Moreover, identification of Tps1 and Nmr1-3 as regulators of CCR re-iterates how carbon and nitrogen metabolism is intimately linked in *M. oryzae*, a likely necessity for its plant pathogenic lifestyle. More work is needed to understand the dynamics of the Nmr inhibitor proteins with their targets, and the identity of those targets. In nitrogen metabolism, all three Nmr proteins converge on Nut1 to repress its activity (including Nmr2 which was not shown to physically interact with Nut1 in yeast two-hybrid studies) [23], whereas the work presented here suggests that in carbon metabolism, each Nmr protein might repress different co-activators of CCR such that taking out any one of the Nmr proteins alleviates repression of its cognate target and activates CCR. Determining the identity of these targets is important because, similarly, deleting any one of the *NMR* genes in Δ*tps1* strains restores virulence [23], suggesting the targets of Nmr1-3 in CCR and in pathogenicity might be similar. More work is needed to identify these targets of Nmr1-3, and Co-IP pull-down experiments will be conducted to identify them. In the future we intend to continue our identification of other components of CCR, and will also undertake the functional characterization of a likely *M. oryzae* homologue of CreA, MGG_11201. *creA^−^* mutant strains were not isolated in our *Agrobacterium*-mediated mutagenesis screen, and targeted deletion of this gene will be undertaken to determine its role in glucose metabolism and infection in *M. oryzae*. In addition, in the event deletion of MGG_11201 is lethal, we will also attempt a gene silencing approach to eliminate MoCreA from different points in the fungal lifecycle. Intriguingly, MGG_11201 gene expression appears under Tps1-control (Hartline and Wilson, unpublished results) and we intend to explore the relationship between Tps1 and MGG_11201 in the future.

Selecting for extragenic suppressors of our available mutant strains, we determined that a MATE-family efflux protein, Mdt1, is an additional regulator of CCR that is necessary for sporulation and essential for virulence. This is the first time a MATE-family protein has been characterized in either a filamentous fungus or a plant pathogen. In addition to identifying a novel pathogenicity factor, this is also the first study to assign a genetic regulatory role to a MATE-family efflux protein. Understanding the function of MATE proteins is clinically important due to their role in multi drug resistance, where bacterial MATE transporters reduce the efficacy of antibiotic treatments by extruding those drugs that resemble native substrates [44], [64], [65]. MATE proteins also influence the pharmacokinetics of therapeutic drug regimes in a similar manner [66]–[68], for instance by affecting the treatment of diabetes through the extrusion of the glucose-lowering drug Metformin [69]. The work described here could serve as a model for understanding the physiological role of these transporters, thus helping to identify their native substrates and contributing to a better understanding of how treatments impacted by MATE proteins could be improved. Moreover, the essential role of Mdt1, a putative transmembrane pump, in plant pathogenesis and sporulation makes it a superb and accessible target for future anti-rice blast strategies.

**Figure 14:**
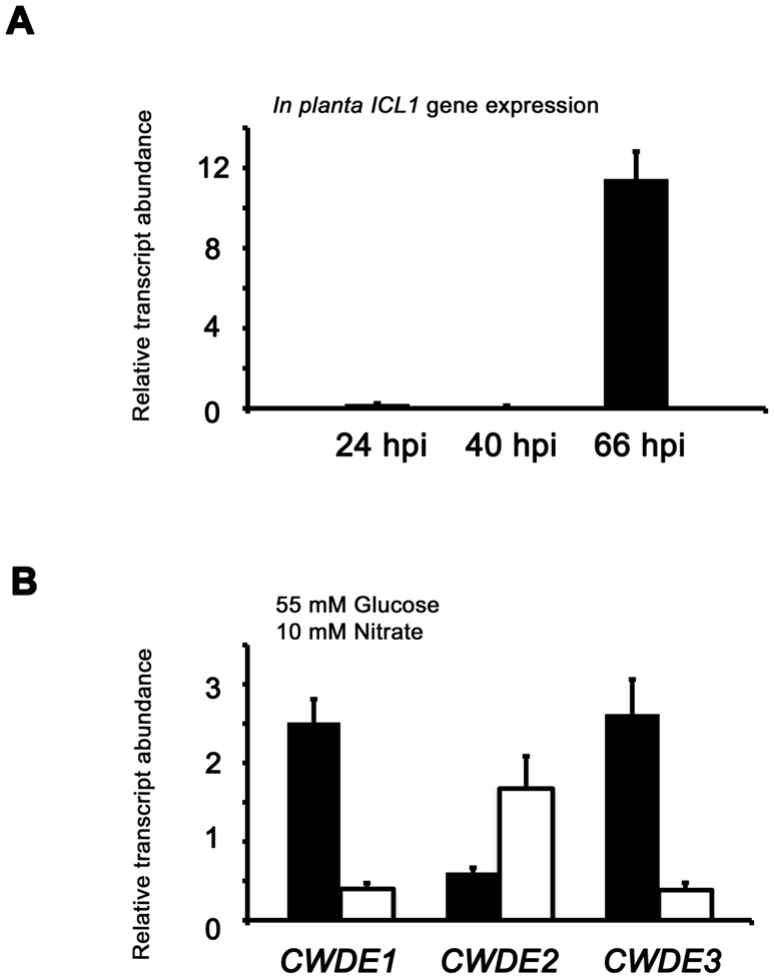
Transcript analysis supports a role for CCR as an important regulator of gene expression during rice infection. (A) During plant infection by Guy11, *ICL1* encoding isocitrate lyase is expressed late in infection during the necrotic stage of disease. *ICL1* expression was monitored at 24, 40 and 66 hpi and shown to be highly expressed after necrotic lesions had developed. Due to cross-reactivity between fungal and rice ß-tubulin orthologues, gene expression results were normalized against expression of the *M. oryzae* actin gene (*ACT1*). Results are the average of at least three independent replicates, and error bars are the standard deviation. (B) CWDE gene expression is altered in Δ*mdt1* mutant strains (open bars) compared to Guy11 (closed bars) following growth on CM for 48 hr followed by a switch to minimal media with 55 mM glucose and 10 mM NO_3_^−^ for 16 hr. *CWDE1* encodes ß-glucosidase 1, *CWDE2* encodes feruloyl esterase B and *CWDE3* encodes exoglucanase. Gene expression results were normalized against expression of the ß-tubulin gene (*TUB2*). Results are the average of at least three independent replicates, and error bars are the standard deviation.

Fungi posses sensitive gene regulatory mechanisms for responding to nutrient fluctuations in the environment, but until recently little was known about these systems in pathogens such as the devastating rice blast fungus *M. oryzae*. Such mechanisms must be essential in *M. oryzae* for three reasons: they would signal the transition of the fungus from the nutrient-free surface to the sugar-rich interior of the host; they would allow the fungus to respond rapidly to the nutritional status of the host; and they would temper the voracious appetite of *M. oryzae* during the biotrophic growth stage in the plant. *M. oryzae* can utilize a wide range of carbon sources in plate tests ([20]; Quispe and Wilson, unpublished), but *in planta* growth is rigorously controlled and choreographed during the early stages of infection, with the fungus residing in one cell for 8–12 hr before moving to the next in a biotrophic and symptomless manner [18]. Only later does the fungus enter its necrotic phase, causing plant tissue destruction and escape of the fungal spores from the host. From our data it is likely CCR contributes to the spatial and temporal regulation of *M. oryzae* development during infection. The work described here suggests a scenario whereby Tps1 and Mdt1 regulate CCR to optimize growth under the changing glucose conditions likely found during ramification throughout the epidermal and mesophyl layer; during the leaf photosynthetic cycle; and during the necrotic phase when leaf cells are destroyed, photosynthesis ceases and G6P levels drop. Figure 14A demonstrates that controling CCR is relevant to the infection process because *ICL1*, which is misregulated in Δ*tps1* and Δ*mdt1* strains, is not expressed by the wild type until the appearance of necrotic lesions. CCR control of CWDEs is also likely to be important for the pathogenicity of *M. oryzae*, and Figure 14B shows that Δ*mdt1* strains, like Δ*tps1* strains, are misregulated for *CWDE* gene expression. Analysis of the genome of the obligate biotrophic plant pathogens *Ustilago maydis* [70] and *Blumeria graminis* [71] reveal they carry a marked reduction in genes encoding CWDEs compared to other plant pathogens, suggesting CWDEs are not required - and may be detrimental - to the biotrophic lifestyle. Therefore, we propose misregulation of CCR in Δ*tps1* and *MDT1* disruption strains during the early biotrophic stages of infection is likely to have profound effects on the ability of *M. oryzae* to establish disease – perhaps in part due impaired glucose assimilation and the perturbed expression of CWDEs.

Identifying the interplay of regulatory systems that condition *M. oryzae* nutrient acquisition and growth in the plant, and how that control can be perturbed, is an ongoing future goal of our research.

## Materials and Methods

### Strains, physiological tests, and plant infections

All strains used in this study were derived from Guy11 (Table S4). Strains were grown on complete medium (CM) containing 1% (W/V) glucose, 0.2% (W/V) peptone, 0.1% (W/V) yeast extract and 0.1% (W/V) casamino acids, or on minimal medium (MM) containing 1% glucose and 0.6% sodium nitrate, unless otherwise stated, as described in [21]. 55 mm petri dishes were used unless stated otherwise. Allyl alcohol (ACROS organics, USA), kanamycin (Fisher, USA), sorbose (Sigma, USA), 2-deoxyglucose (Sigma, USA) and ethionine (Sigma, USA) were added to CM or MM in the amounts indicated. Plate images were taken with a Sony Cyber-shot digital camera, 14.1 mega pixels. Nitrate reductase enzyme activity was measured as described previously [21]. For spore counts, 10 mm^2^ blocks of mycelium were transferred to the centre of each plate, and the strains grown for 12 days at 26°C with 12 hr light/dark cycles. Spores harvested in sterile distilled water, vortexed vigorously and counted on a haemocytometer (Corning). Spores were counted independently at least four times. Rice plant infections were made using a susceptible dwarf Indica rice (*Oryza sativa*) cultivar, CO-39, as described previously [23]. Fungal spores were isolated from 12–14 day-old plate cultures and spray-inoculated onto rice plants of cultivar CO-39 in 0.2% gelatin at a concentration of 5×10^4^ spores/ml, unless otherwise stated, and disease symptoms were allowed to develop under conditions of high relative humidity for 96–144 hrs.

### Gene transcript analysis

For fungal gene transcript studies, strains were grown for 48 h in CM before switching to minimal media for 16 hr, unless otherwise stated. Mycelia was harvested, frozen in liquid nitrogen, and lyophilised overnight. For leaf RNA extractions, tissues were weighed and approximately 100 mg of tissue was frozen in liquid nitrogen and ground in a mortar and pestle. RNA was extracted from fungal mycelium and infected leaf tissue usng the RNeasy mini kit from Qiagen. RNA was converted to cDNA using the qScript reagents from Quantas. Real time quantitative PCR was performed on an Eppendorf Mastercycler Realplex using the recommended reagents with primers designed using the netprimer software program (Table S5). qPCR data was analyzed using the Realplex software. Thermocycler conditions were: 10 min at 95°C, followed by 40 cycles of 95°C for 30 sec, 63°C for 30 sec and 72°C for 30 sec.

### *Agrobacterium*-mediated transformation of *Magnaporthe oryzae*

*A. tumefaciens-*mediated transformation was performed as previously described [72] by incubating the *Agrobacterium* strain ALG1 containing the binary vector pKHt [40] with 0.5–1.0 g of fungal mycelia grown as a liquid shake in CM.

Proline and glucosamine selection plates were prepared by first pouring a 3–5 mm thick support layer of minimal media without any carbon or nitrogen source. Celluose nitrate membranes containing co-incubated *Agrobacterium* and *Magnaporthe* strains were laid on top of this support layer and the metabolic selection was then poured over the co-incubation membranes. The selection media contained glucose with proline or glucosamine plus 250 µg/ml hygromycin (CalBiochem), 400 µg/ml cefotaxime (Research Products International Corp), 100 µg/ml carbenicillin (Fisher BioReagents), and 60 µg/ml streptomycin (Fisher BioReagents). These antibiotics both kill the *Agrobacterium* and select for hygromycin insertion. Following standard incubation conditions, colonies appeared in 5–10 days and were transferred to a purification plate containing the appropriate carbon and nitrogen sources and the antibiotics described above.

To identify which gene was mutated by T-DNA insertion, DNA was extracted from purified colonies as described previously [73]. DNA sequences flanking the right border of the T-DNA inserts were amplified by inverse PCR [74]. Genomic DNA was digested with *BamHI* (Fermentas), ligated to circularize the products using T4 DNA Ligase (NEB), and amplified by PCR using primers designed from the known sequence of the *Hph* gene, conferring hygromycin resistance, present in the T-DNA insert (Table S5). PCR conditions were 1 min at 95°C followed by 35 cycles of 30 sec at 95°C, 30 sec at 63°C and 3 min at 68°C. PCR products were subcloned into pGEM-T (Promega), transformed into JM109 competent cells (Promega) and sequenced by Eurofins MWG Operon, USA.

### Genetic manipulations

Targeted gene replacement was achieved by the split marker method described in [23] using the oligonucleotide primers shown in Table S5. Δ*nut1* was generated in Guy11 and Δ*tps1* parental strains using the *ILV1* gene conferring sulphonyl urea resistance as the selectable marker. *MDT1* was deleted in all the strains studied using the *bar* gene conferring bialaphos resistance. The hexose phoshorylase genes *HXK1*, *HXK2 and GLK1* were deleted in Guy11 using the *bar* gene conferring bialaphos resistance resistance as the selectable marker. *ZAP1* was deleted in Guy11 using the *ILV1* gene conferring sulphonyl urea resistance as the selectable marker. Gene deletions were verified by PCR as described previously [23]. The role of *MDT1* in pathogenicity was verified by complementation through the introduction of a plasmid carrying *MDT1* into the Δ*mdt1* deletion strain. Resulting complementation strains were tested for pathogenicity on rice leaves. The full length *MDT1* complementation vector was constructed usng the primer pairs shown in Table S5 and following the protocol of Zhou et al [75].

### Protein extraction and LC/MS/MS analysis

Proteomic studies were performed as follows. Strains were grown in CM for 48 hrs, then transferred to minimal media with nitrate for 16 hrs, following [23]. 500 mg of fungal biomass (wet weight) was transferred in to a 1 ml lysis buffer comprising 8 M urea in 100 mM ammonium bicarbonate and containing 1.5 mM protease inhibitor (PMSF, Sigma). The biomass was then subjected to bead beating using a glass bead beater for 3 minutes at 4°C. The supernatant was collected after centrifugation at 10000 g for 20 minutes. The protein in the supernatant was collected by cold acetone precipitation (1 ml of sample added with 9 ml of acetone) overnight at −2°C. The resultant precipitate was collected by centrifugation at 7000 g for 30 minutes. The precipitate was air dried to remove residual acetone and the dried pellets were resolubilised in 250 µl of 100 mM ammonium bicarbonate. The protein concentration was estimated using the BCA protein assay kit (Thermo Fisher Scientific).

For the LC/MS/MS experiments, in solution trypsin digestion was performed on the extracted fungal protein using the protocol described in [76]. Following 16 hours of tryptic digestion the reaction was terminated by adding 0.1% formic acid. The peptide solution was dried using a speed vacuum drier (Thermo fisher) and reconstituted with 20 µl of 0.1% formic acid in water. These peptides were later subjected to LC/MS/MS analysis with an ultimate 3000 Dionex nano LC system (Dionex corporation) integrated with LCQ Fleet Ion Trap mass spectrometer (Thermo scientific) equipped with a nano source. The acquired MS/MS spectrum was searched against the *Magnaporthe oryzae* protein sequence database (NCBI) using MASCOT (Matrix Sciences, UK) bioinformatics software to identify the protein and Scaffold software (Proteome Software Inc., USA) for further spectrum counting and relative protein quantification analysis.

### Metabolite analysis

The citrate in the fungal culture broth was measured using liquid chromatography mass spectrometry in SRM mode (Single reaction mode). 10 µl of the centrifuged culture broth sample was injected into the LC-MS/MS system. An LC system (Agilent 1200 series HPLC) was integrated with a C18 column (50×2.1 mm, Thermo Fisher GOLD) with a flow rate of 0.3 ml/min water containing 2 mmol l^−1^ ammonium acetate and 0.1% (v/v) formic acid for loading the samples. A step gradient of 100% Acetonitrile containing 2 mmol l^−1^ ammonium acetate and 0.1% (v/v) formic acid was used to wash the column. The citrate eluted from the column isocratically with a retention time of 0.52 min. The analytes were monitored using a triple quadruple mass spectrometer (AB SCIEX Q Trap 4000) operated in multiple reaction monitoring mode using the following transition: citrate m/z 193.10>175.0. An external calibration was set using the same conditions using pure citrate (Sigma, USA) and peak area was calculated for quantitation purpose using the Analyst 1. 5.1 software (AB SCIEX). The unknown concentration of the citrate was calculated from the obtained calibration curve.

## Supporting Information

Figure S1 Tps1 regulates nitrogen metabolism and pathogenicity. In response to G6P sensing, Tps1 inhibits the Nmr inhibitor proteins (Nmr1, Nmr2, Nmr3) by elevating NADPH levels. This is postulated to alleviate Nmr inhibition of at least three GATA factors, Pas1 (a White Collar-2 homologue), Asd4 (essential for appressorium development and pathogenicity) and Nut1 (dispensable for pathogenicity but required for nitrogen metabolism) [23]. Nmr represents the Nmr1, Nmr2 and Nmr3 inhibitor proteins. GATA represents the Pas1, Asd4 and Nut1 GATA family transcription factors.
(TIF)

Figure S2 Plate tests of Guy11 and Δ*nut1* strains on different carbon and nitrogen sources. (A) Other than growth on minimal media containing 10 mM glucose and 10 mM proline, the Δ*nut1* strains generated in this study conform to the nitrogen source utilization phenotype described by Froeliger and Carpenter [22]. This includes no growth on nitrate or nitrite as sole nitrogen sources, but good growth on ammonium, glutamate and alanine as nitrogen sources. CM is complete media. All other plates are minimal media supplemented with 10 mM of the appropriate carbon and nitrogen source. (B) 10 mM aminoisobutyric acid can be used as a nitrogen source but not a carbon source by the *M. oryzae* wild type strain Guy11.
(TIF)

Figure S3 Plate tests to assay for carbon derepression. (A) Guy11, Δ*tps1*, Δ*tps1::R22G* and Δ*tps1::Y99V* strains and (B) Guy11, Δ*glk1*, Δ*hxk1* and Δ*hxk2* strains, were grown on defined minimal media with 55 mM (ie 1%) glucose and 10 mM NH_4_^+^ as sole carbon and nitrogen source, respectively, with or without supplementation by 100 mM of the toxic analogue allyl alcohol (AA). Strains were grown for 5 days on 85 mm petri dishes, and radial diameters were measured. The diameters of strains grown on minimal media+100 mM AA are given as a percentage of the diameters of the same strains grown on minimal media only. Results are the average of three independent replicates. Error bars are standard deviation. Bars with the same letters are not significantly different (*Student's t-test* p≤0.01).
(TIF)

Figure S4 qPCR analysis of Tps1-dependent gene expression. Gene expression results were normalized against expression of the ß-tubulin gene (*TUB2*). Results are the average of at least three independent replicates, and error bars are the standard deviation. (A) The expression of three genes encoding the putative glucose transporters *GHT2*, *RGT2* and *HXT1* were analyzed in strains of Guy11 (black bars) and Δ*tps1* (open bars). Strains were grown in CM media for 48 hr before switching to 55 mM glucose+10 mM NO_3_^−^ minimal media for 16 hr. (B) qPCR analysis of hexose kinase gene expression in Guy11 (black bars) and Δ*tps1* (open bars) shows that *HXK1*, *HXK2* and *GLK1* expression is Tps1-dependent. Strains were grown in CM media for 48 hr before switching to 55 mM glucose+10 mM NO_3_^−^ minimal media for 16 hr. (C) *GHT2* gene expression was analyzed in Guy11 strains following growth on CM for 48 hr followed by a switch to minimal media with 55 mM glucose and 10 mM NH_4_^+^ (black bar) or minimal media with 55 mM glucose and no nitrogen source (grey bar). (D and E) Guy11 (closed bars) and Δ*tps1* strains (open bars) were grown in CM media for 48 hr before switching to 55 mM glucose+10 mM NO_3_^−^ minimal media for 16 hr. Tps1 is required for repressing proline (*PRN3*) glucosamine (*GNI1*) and alcohol (*ADH1*) metabolic gene expression during growth on glucose-containing minimal media. (F) To determine if internal proline was carried over from the CM media, strains were grown in CM for 48 hr followed by a switch to minimal starvation media lacking a carbon and nitrogen source for 12 hr followed by a second switch to minimal media with 55 mM glucose and 10 mM NO_3_^−^. *PRN3* gene expression was analyzed in Guy11 and Δ*tps1* strains following these treatments and was significantly elevated in Δ*tps1* strains (*Student's t-test* p≤0.01) compared to wild type.
(TIF)

Figure S5 Deletion of Δ*nmr1* in the Δ*tps1* deletion strain partially restores virulence compared to Guy11. Spores were applied to rice leaves at a rate of 1×10^4^ ml^−1^. Δ*tps1* strains are non-pathogenic. After 72 hpi, Δ*tps1* Δ*nmr1* strains developed characteristic eye-spot necrotic lesions that were reduced in size compared to Guy11.
(TIF)

Figure S6 qPCR analysis of genes regulated by the Nmr1-3 inhibitor proteins independently of Nut1 activity. (A) The expression of *HXK2* and (B) *GLK1* was analyzed in Guy11, Δ*nut1*, Δ*tps1*, Δ*tps1* Δ*nmr1*, Δ*tps1* Δ*nmr2*, and Δ*tps1* Δ*nmr3* strains and were shown to be expressed independently of *NUT1* and elevated in expression in Δ*tps1* Δ*nmr1-3* suppressor strains compared to Δ*tps1* strains. (C) *G6PDH* gene expression had previously been shown to be Tps1-dependent and restored to Guy11 levels of expression in Δ*tps1* Δ*nmr1-3* suppressor strains [23]. Here, we show that *G6PDH* gene expression is independent of *NUT1*. For (A)–(C), strains were grown in CM media for 48 hr before switching to 55 mM glucose+10 mM NO_3_^−^ minimal media for 16 hr. This media was chosen to determine if CCR-dependent gene expression is independent of Nut1. Gene expression results were normalized against the expression of the ß-tubulin gene (*TUB2*) and given relative to the expression of each gene in Guy11. Results are the average of at least three independent replicates, and error bars are the standard deviation.
(TIF)

Figure S7 Characterizing *NIA1* gene expression. (A) *NIA1* gene expression was analyzed in Guy11 strains in the presence and absence of an inducer or a carbon source. Guy11 was grown in CM media for 48 hr before switching to 55 mM glucose (Glc)+10 mM NO_3_^−^ minimal media, minimal media with 10 mM NO_3_^−^ but without a source of carbon (-C), minimal media containing 55 mM glucose but no nitrogen source (-N), or nitrogen repressing minimal media containing 55 mM glucose and 10 mM NH_4_^+^, for 16 hr. Gene expression results were normalized against the expression of the ß-tubulin gene (*TUB2*) and given relative to the expression of *Nia1* in Guy11 on 55 mM glucose+10 mM NO_3_^−^ minimal media. Results are the average of at least three independent replicates, and error bars are the standard deviation. (B) RNA was extracted from appressoria of Guy11 and Δ*tps1* strains as described previously [23]. Gene expression results were normalized against the expression of the ß-tubulin gene (*TUB2*). Results are the average of at least three independent replicates, and error bars are the standard deviation.
(TIF)

Figure S8 T-DNA insertion and homologous gene replacement of *MDT1* in Δ*nut1* strains. (A) *A. tumefaciens*-mediated transformation using the binary vector pKHt [40] was performed to randomly insert T-DNA into the genome of Δ*nut1* parental strains. Extragenic suppressor strains that restored the growth of Δ*nut1* on glucosamine or proline as nitrogen source were determined, using inverse PCR, to result from T-DNA insertion into the 3′ coding region of *MDT1*. (B) To functionally characterize *MDT1*, and to confirm T-DNA insertion resulted in disruption of the *MDT1* gene, the 3′ end of *MDT1* was replaced with the *Bar* gene conferring resistance to bialaphos using the split marker strategy for homologous gene replacement [23].
(TIF)

Figure S9 Exploring Mdt1 function. (A) To determine if the Mdt1 efflux protein has a role in ethionine resistance, strains were grown on minimal media with 10 mM glucose and 10 mM NH_4_^+^ (left panel) or the same media supplemented with 50 µg/L ethionine. Even at the relatively high concentrations of ethionine shown, some growth was observed for *MDT1* deletion strains, suggesting deletion of *MDT1* did not make *M. oryzae* more susceptible to ethionine. (B) To determine if loss of Mdt1 function renders strains defective in glucose uptake, Guy11, Δ*nut1*, d*nut1 Supp 3121022*, Δ*nut1* Δ*mdt1* and Δ*mdt11* strains were grown for 10 days on 85 mm petri-dishes containing carbon derepressing minimal media consisting of 10 mM xylose+10 mM NH_4_^+^ as sole carbon and nitrogen sources and the same media supplemented with 5 mM sorbose or 50 µg/mL 2-deoxyglucose (2-DOG). Strains with disrupted Mdt1 function were not more resistant to sorbose or 2-DOG compared to Guy11, suggesting glucose uptake and/or phosphorylation is not significantly impaired in these strains. (C) Mdt1 does not confer resistance to reactive oxygen species. Strains were grown for 10 days on CM supplemented with 10 mM H_2_O_2_.
(TIF)

Figure S10 The role of zinc metabolism in rice blast disease. (A) *ZAP1* encodes a putative zinc finger protein with a role in zinc acquisition. Guy11 and Δ*zap1* strains were grown on minimal media containing the standard zinc concentration (1×Zn) or 100-fold less zinc (1∶100×Zn). Reduced sporulation of Δ*zap1* on 1∶100×Zn GMM indicates *ZAP1* has a role in zinc homeostasis and acquisition. Spores were harvested from plates following 12 days of growth. Values are the average of at least three independent replicates and bars are standard error. Bars with the same letters are not significantly different (*Student's t-test* p≤0.05). (B) *ZAP1* is not required for infection. Guy11 and Δ*zap1* strains were inoculated at a rate of 1×10^5^ spores/ml. No loss of pathogenicity was observed in Δ*zap1* strains indicating reduced zinc uptake from the plant is not an impediment to disease establishment.
(TIF)

Table S1 Description of *Magnaporthe oryzae* genes analysed in this study.
(DOC)

Table S2 The comparative proteome of Δ*tps1* and Guy11 mycelial samples following growth on glucose minimal media.
(XLS)

Table S3 Extragenic suppressors of Δ*nut1* strains generated by *Agrobacterium tumefaciens*-mediated mutagenesis.
(DOCX)

Table S4 *Magnaporthe oryzae* strains used in this study.
(DOCX)

Table S5 Oligonucleotide primers used in this study.
(DOC)
