# Supplementary figures and images for: Principles of Carbon Catabolite Repression in the Rice Blast Fungus: Tps1, Nmr1-3, and a MATE–Family Pump Regulate Glucose Metabolism during Infection
Source: PLoS Genet. 2012 May 3;8(5):e1002673. doi: 10.1371/journal.pgen.1002673 (PMC3342947; doi:10.1371/journal.pgen.1002673)

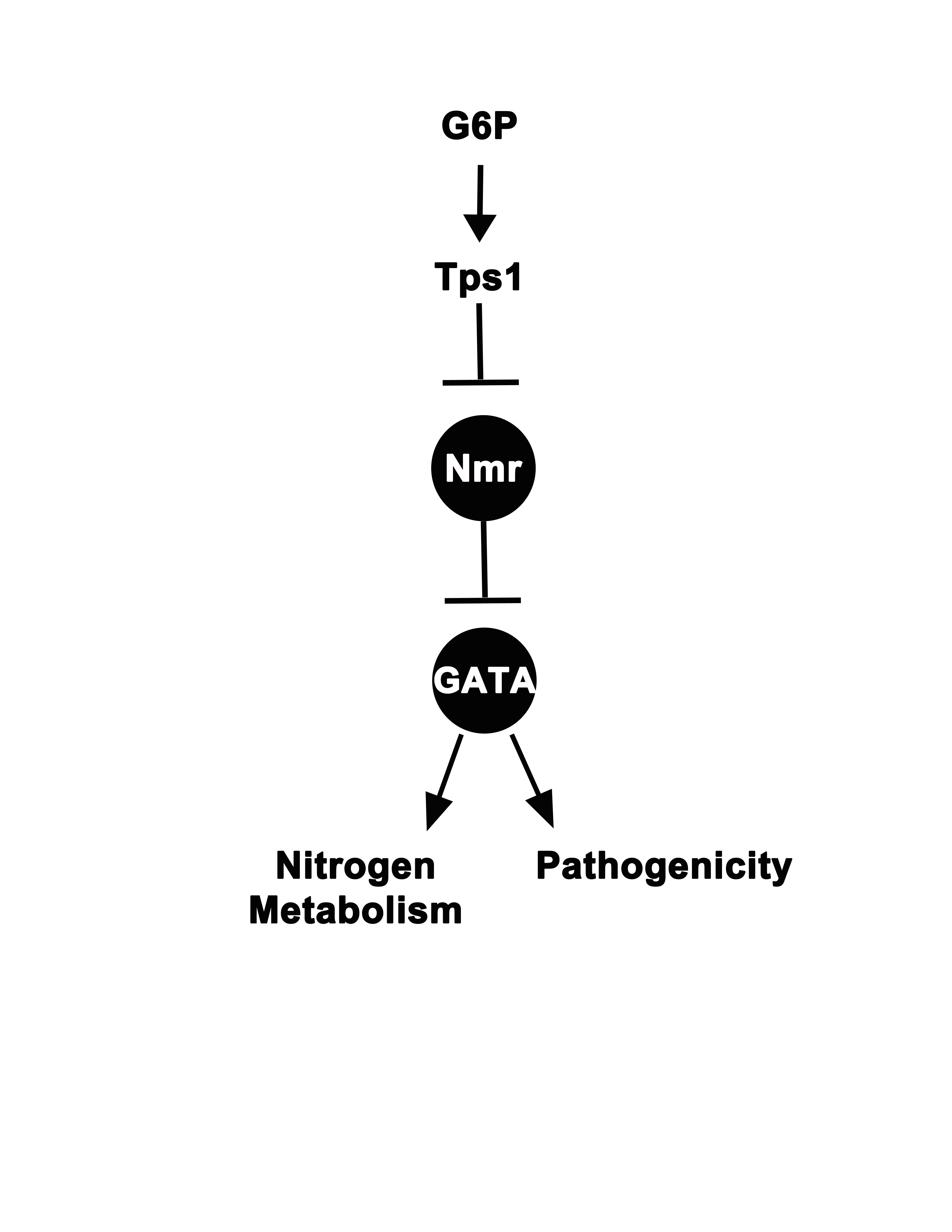

Supplement: Figure S1 — Tps1 regulates nitrogen metabolism and pathogenicity. In response to G6P sensing, Tps1 inhibits the Nmr inhibitor proteins (Nmr1, Nmr2, Nmr3) by elevating NADPH levels. This is postulated to alleviate Nmr inhibition of at least three GATA factors, Pas1 (a White Collar-2 homologue), Asd4 (essential for appressorium development and pathogenicity) and Nut1 (dispensable for pathogenicity but required for nitrogen metabolism) [23]. Nmr represents the Nmr1, Nmr2 and Nmr3 inhibitor proteins. GATA represents the Pas1, Asd4 and Nut1 GATA family transcription factors. (TIF) [file pgen.1002673.s001.tif]

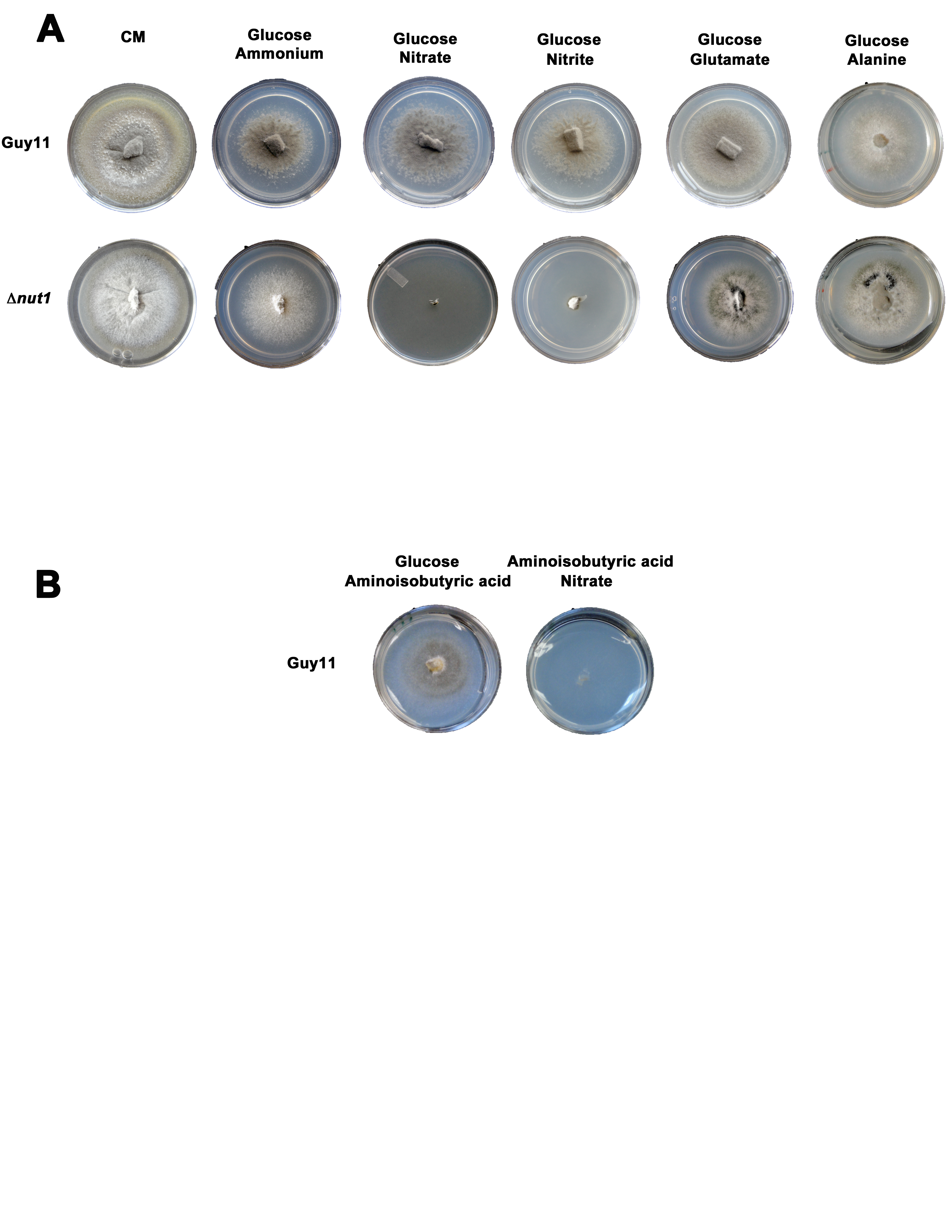

Supplement: Figure S2 — Plate tests of Guy11 and Δnut1 strains on different carbon and nitrogen sources. (A) Other than growth on minimal media containing 10 mM glucose and 10 mM proline, the Δnut1 strains generated in this study conform to the nitrogen source utilization phenotype described by Froeliger and Carpenter [22]. This includes no growth on nitrate or nitrite as sole nitrogen sources, but good growth on ammonium, glutamate and alanine as nitrogen sources. CM is complete media. All other plates are minimal media supplemented with 10 mM of the appropriate carbon and nitrogen source. (B) 10 mM aminoisobutyric acid can be used as a nitrogen source but not a carbon source by the M. oryzae wild type strain Guy11. (TIF) [file pgen.1002673.s002.tif]

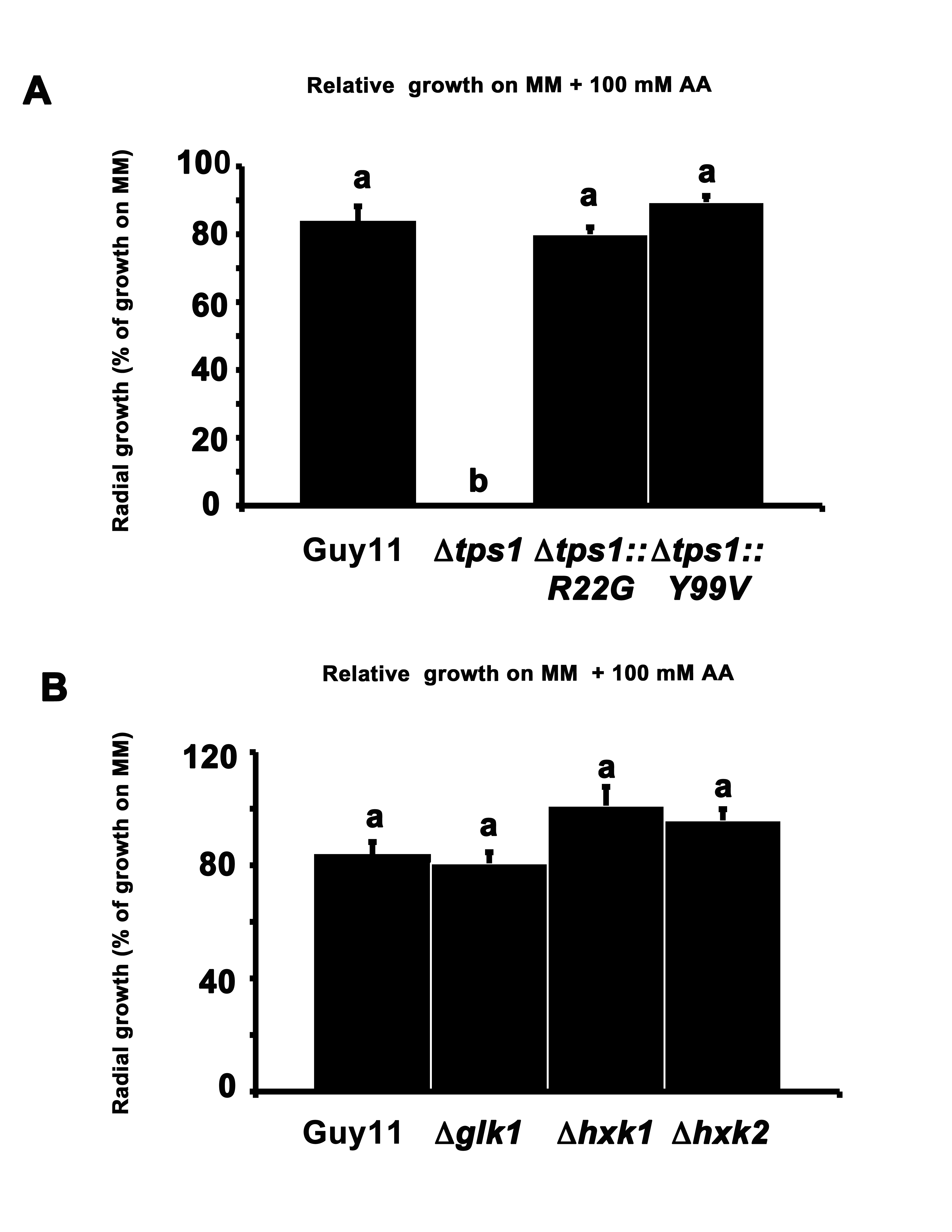

Supplement: Figure S3 — Plate tests to assay for carbon derepression. (A) Guy11, Δtps1, Δtps1::R22G and Δtps1::Y99V strains and (B) Guy11, Δglk1, Δhxk1 and Δhxk2 strains, were grown on defined minimal media with 55 mM (ie 1%) glucose and 10 mM NH4 + as sole carbon and nitrogen source, respectively, with or without supplementation by 100 mM of the toxic analogue allyl alcohol (AA). Strains were grown for 5 days on 85 mm petri dishes, and radial diameters were measured. The diameters of strains grown on minimal media+100 mM AA are given as a percentage of the diameters of the same strains grown on minimal media only. Results are the average of three independent replicates. Error bars are standard deviation. Bars with the same letters are not significantly different (Student's t-test p≤0.01). (TIF) [file pgen.1002673.s003.tif]

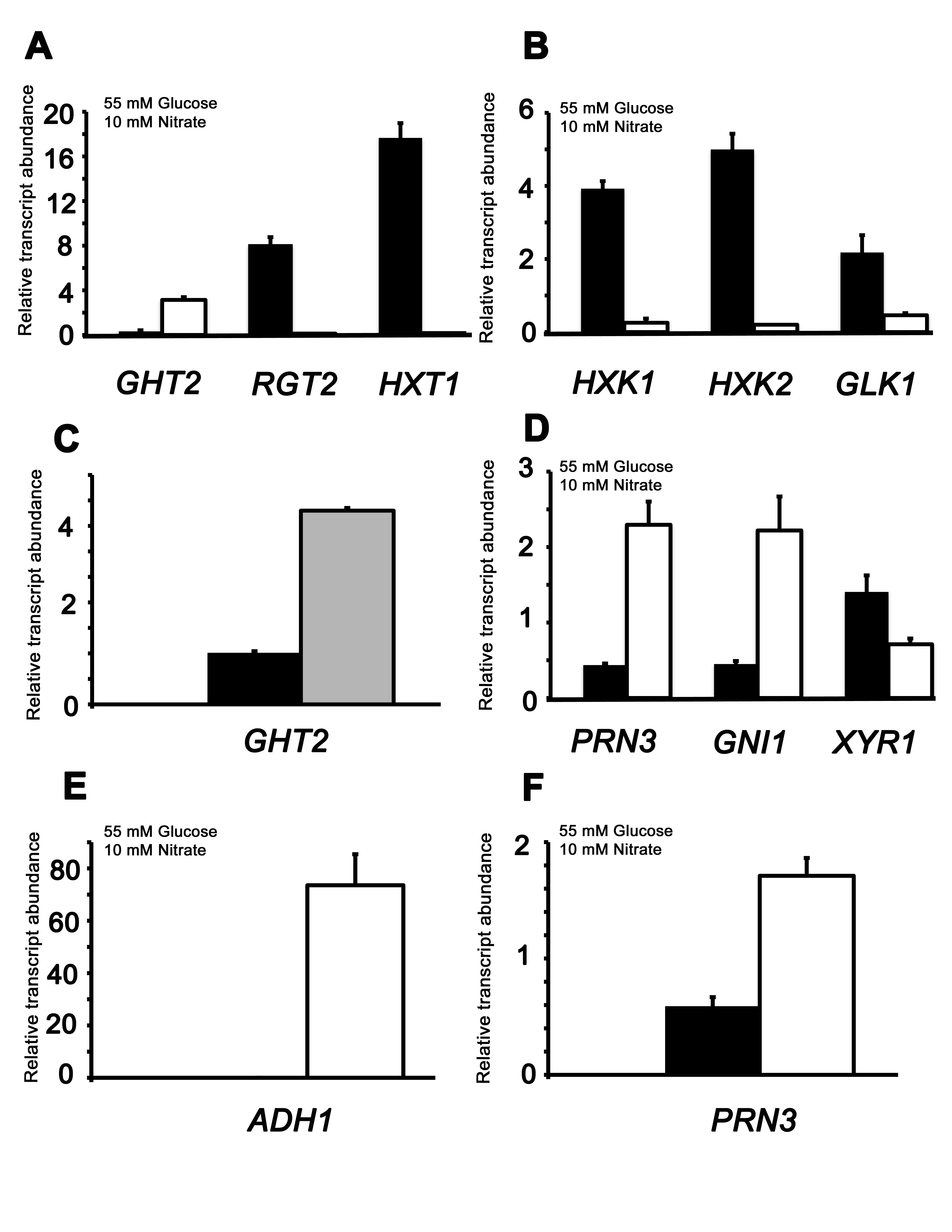

Supplement: Figure S4 — qPCR analysis of Tps1-dependent gene expression. Gene expression results were normalized against expression of the ß-tubulin gene (TUB2). Results are the average of at least three independent replicates, and error bars are the standard deviation. (A) The expression of three genes encoding the putative glucose transporters GHT2, RGT2 and HXT1 were analyzed in strains of Guy11 (black bars) and Δtps1 (open bars). Strains were grown in CM media for 48 hr before switching to 55 mM glucose+10 mM NO3 − minimal media for 16 hr. (B) qPCR analysis of hexose kinase gene expression in Guy11 (black bars) and Δtps1 (open bars) shows that HXK1, HXK2 and GLK1 expression is Tps1-dependent. Strains were grown in CM media for 48 hr before switching to 55 mM glucose+10 mM NO3 − minimal media for 16 hr. (C) GHT2 gene expression was analyzed in Guy11 strains following growth on CM for 48 hr followed by a switch to minimal media with 55 mM glucose and 10 mM NH4 + (black bar) or minimal media with 55 mM glucose and no nitrogen source (grey bar). (D and E) Guy11 (closed bars) and Δtps1 strains (open bars) were grown in CM media for 48 hr before switching to 55 mM glucose+10 mM NO3 − minimal media for 16 hr. Tps1 is required for repressing proline (PRN3) glucosamine (GNI1) and alcohol (ADH1) metabolic gene expression during growth on glucose-containing minimal media. (F) To determine if internal proline was carried over from the CM media, strains were grown in CM for 48 hr followed by a switch to minimal starvation media lacking a carbon and nitrogen source for 12 hr followed by a second switch to minimal media with 55 mM glucose and 10 mM NO3 −. PRN3 gene expression was analyzed in Guy11 and Δtps1 strains following these treatments and was significantly elevated in Δtps1 strains (Student's t-test p≤0.01) compared to wild type. (TIF) [file pgen.1002673.s004.tif]

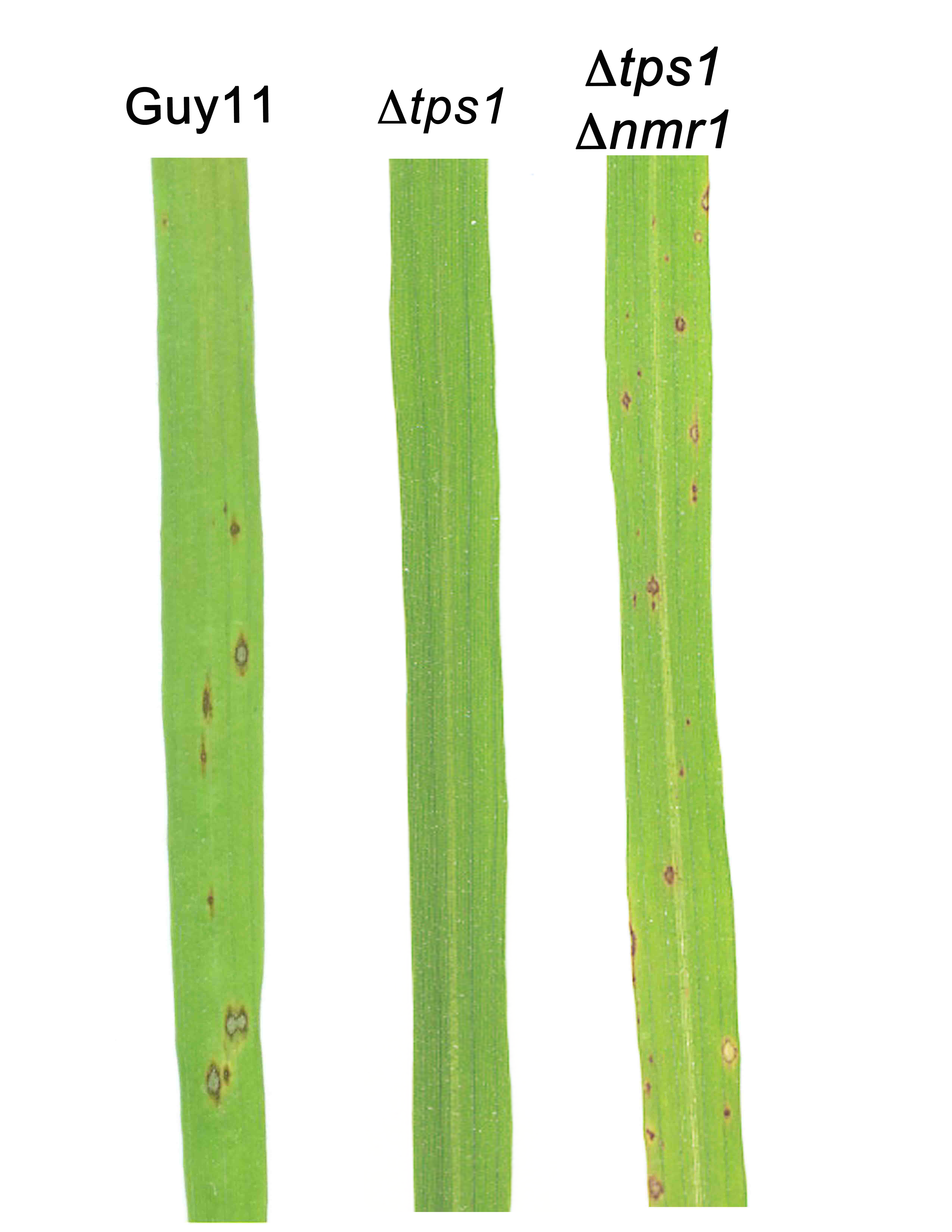

Supplement: Figure S5 — Deletion of Δnmr1 in the Δtps1 deletion strain partially restores virulence compared to Guy11. Spores were applied to rice leaves at a rate of 1×104 ml−1. Δtps1 strains are non-pathogenic. After 72 hpi, Δtps1 Δnmr1 strains developed characteristic eye-spot necrotic lesions that were reduced in size compared to Guy11. (TIF) [file pgen.1002673.s005.tif]

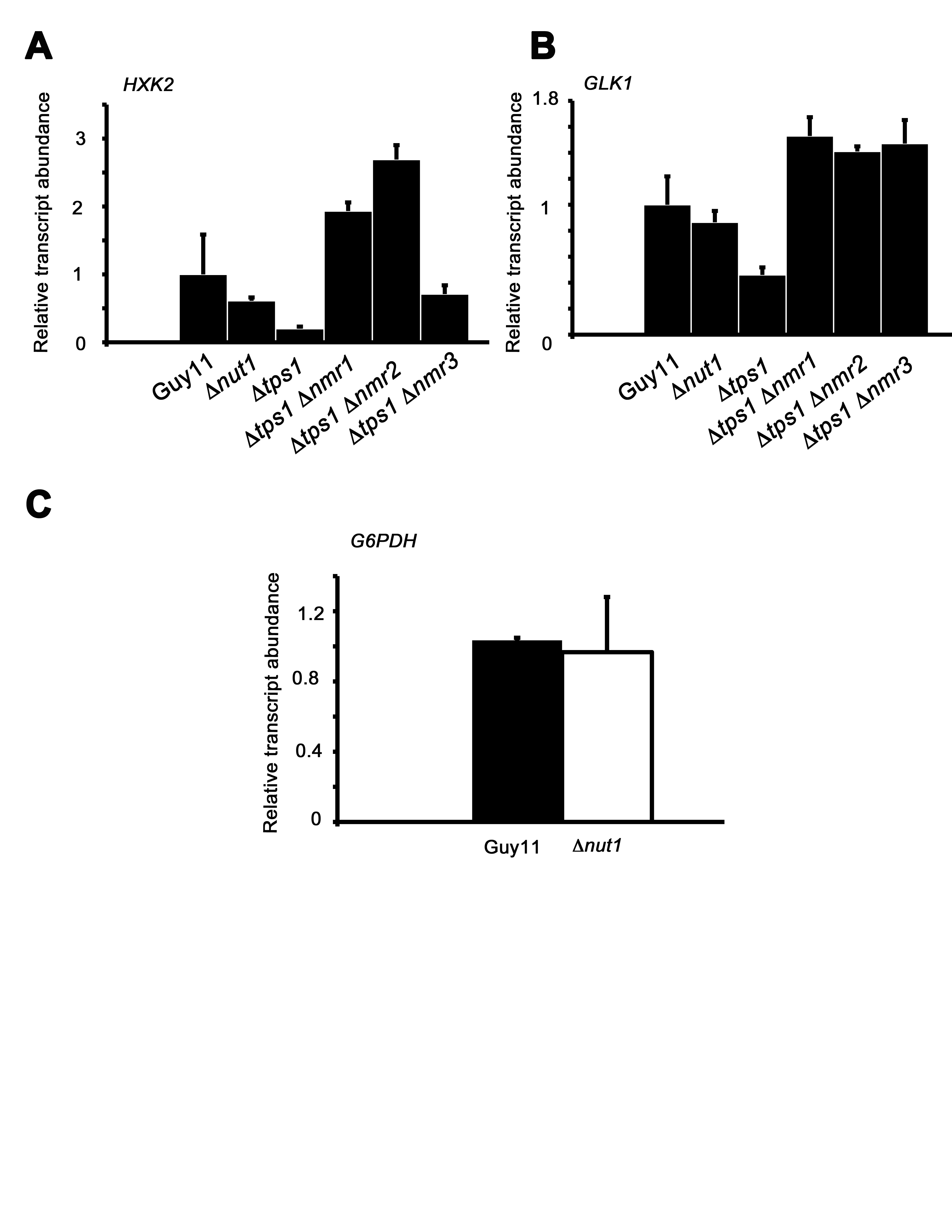

Supplement: Figure S6 — qPCR analysis of genes regulated by the Nmr1-3 inhibitor proteins independently of Nut1 activity. (A) The expression of HXK2 and (B) GLK1 was analyzed in Guy11, Δnut1, Δtps1, Δtps1 Δnmr1, Δtps1 Δnmr2, and Δtps1 Δnmr3 strains and were shown to be expressed independently of NUT1 and elevated in expression in Δtps1 Δnmr1-3 suppressor strains compared to Δtps1 strains. (C) G6PDH gene expression had previously been shown to be Tps1-dependent and restored to Guy11 levels of expression in Δtps1 Δnmr1-3 suppressor strains [23]. Here, we show that G6PDH gene expression is independent of NUT1. For (A)–(C), strains were grown in CM media for 48 hr before switching to 55 mM glucose+10 mM NO3 − minimal media for 16 hr. This media was chosen to determine if CCR-dependent gene expression is independent of Nut1. Gene expression results were normalized against the expression of the ß-tubulin gene (TUB2) and given relative to the expression of each gene in Guy11. Results are the average of at least three independent replicates, and error bars are the standard deviation. (TIF) [file pgen.1002673.s006.tif]

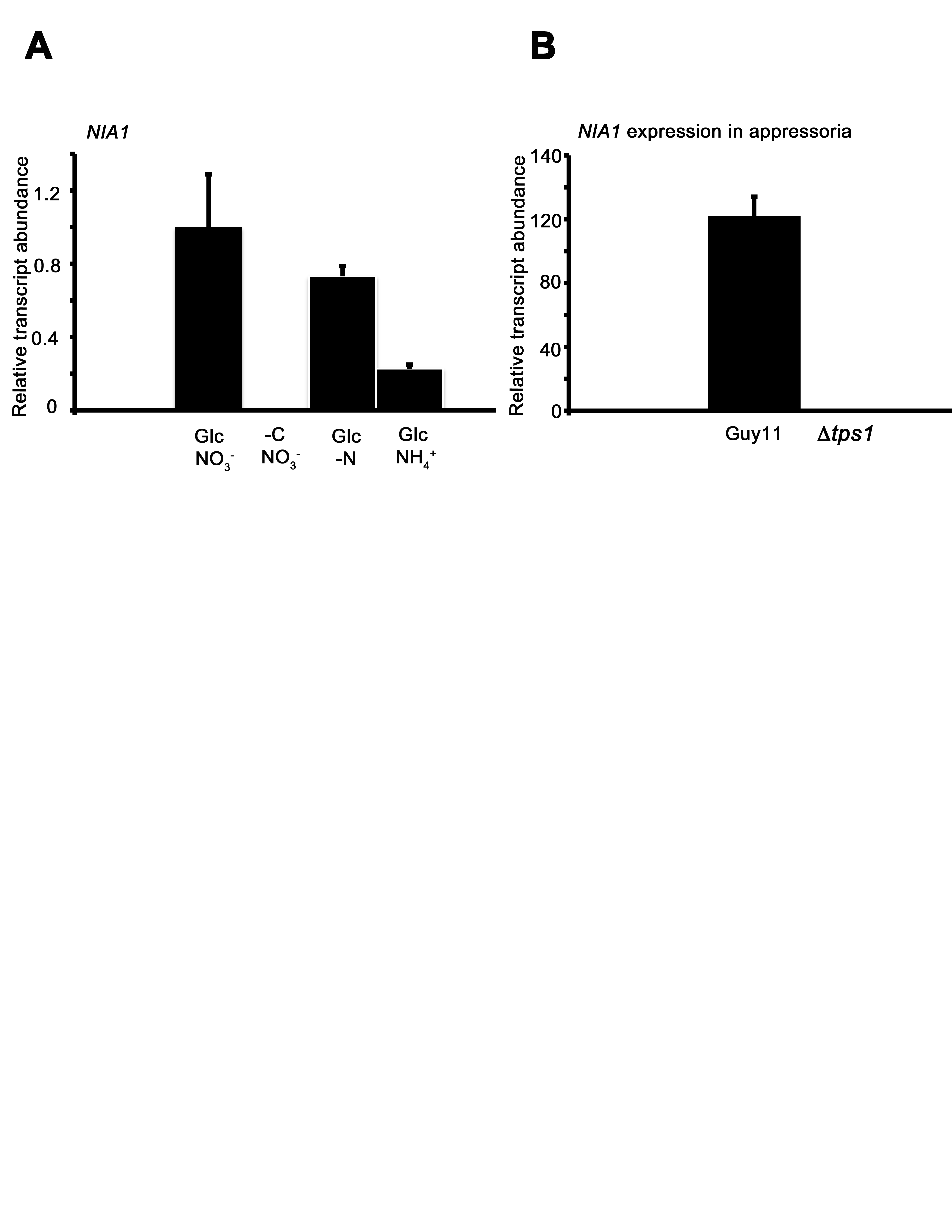

Supplement: Figure S7 — Characterizing NIA1 gene expression. (A) NIA1 gene expression was analyzed in Guy11 strains in the presence and absence of an inducer or a carbon source. Guy11 was grown in CM media for 48 hr before switching to 55 mM glucose (Glc)+10 mM NO3 − minimal media, minimal media with 10 mM NO3 − but without a source of carbon (-C), minimal media containing 55 mM glucose but no nitrogen source (-N), or nitrogen repressing minimal media containing 55 mM glucose and 10 mM NH4 +, for 16 hr. Gene expression results were normalized against the expression of the ß-tubulin gene (TUB2) and given relative to the expression of Nia1 in Guy11 on 55 mM glucose+10 mM NO3 − minimal media. Results are the average of at least three independent replicates, and error bars are the standard deviation. (B) RNA was extracted from appressoria of Guy11 and Δtps1 strains as described previously [23]. Gene expression results were normalized against the expression of the ß-tubulin gene (TUB2). Results are the average of at least three independent replicates, and error bars are the standard deviation. (TIF) [file pgen.1002673.s007.tif]

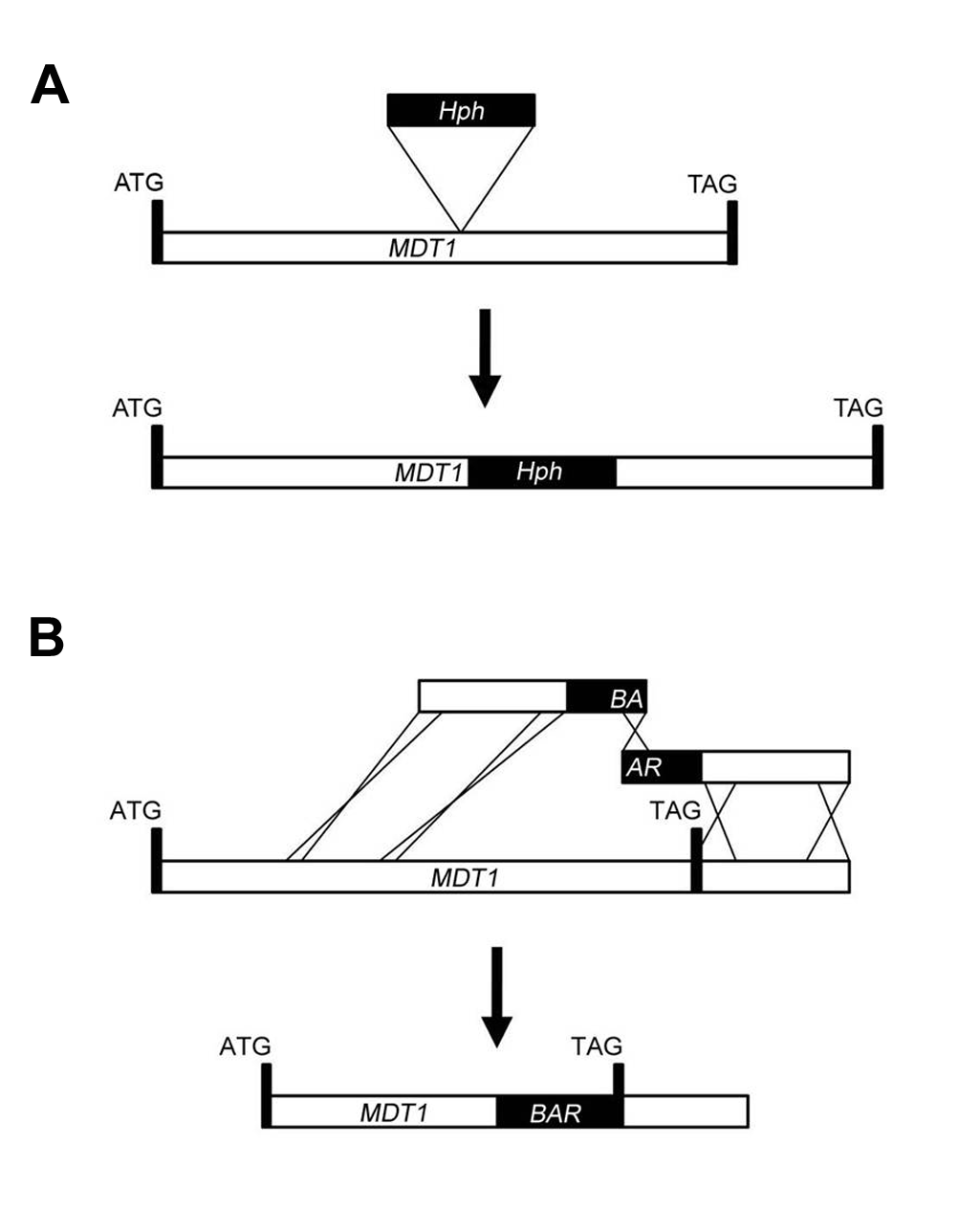

Supplement: Figure S8 — T-DNA insertion and homologous gene replacement of MDT1 in Δnut1 strains. (A) A. tumefaciens-mediated transformation using the binary vector pKHt [40] was performed to randomly insert T-DNA into the genome of Δnut1 parental strains. Extragenic suppressor strains that restored the growth of Δnut1 on glucosamine or proline as nitrogen source were determined, using inverse PCR, to result from T-DNA insertion into the 3′ coding region of MDT1. (B) To functionally characterize MDT1, and to confirm T-DNA insertion resulted in disruption of the MDT1 gene, the 3′ end of MDT1 was replaced with the Bar gene conferring resistance to bialaphos using the split marker strategy for homologous gene replacement [23]. (TIF) [file pgen.1002673.s008.tif]

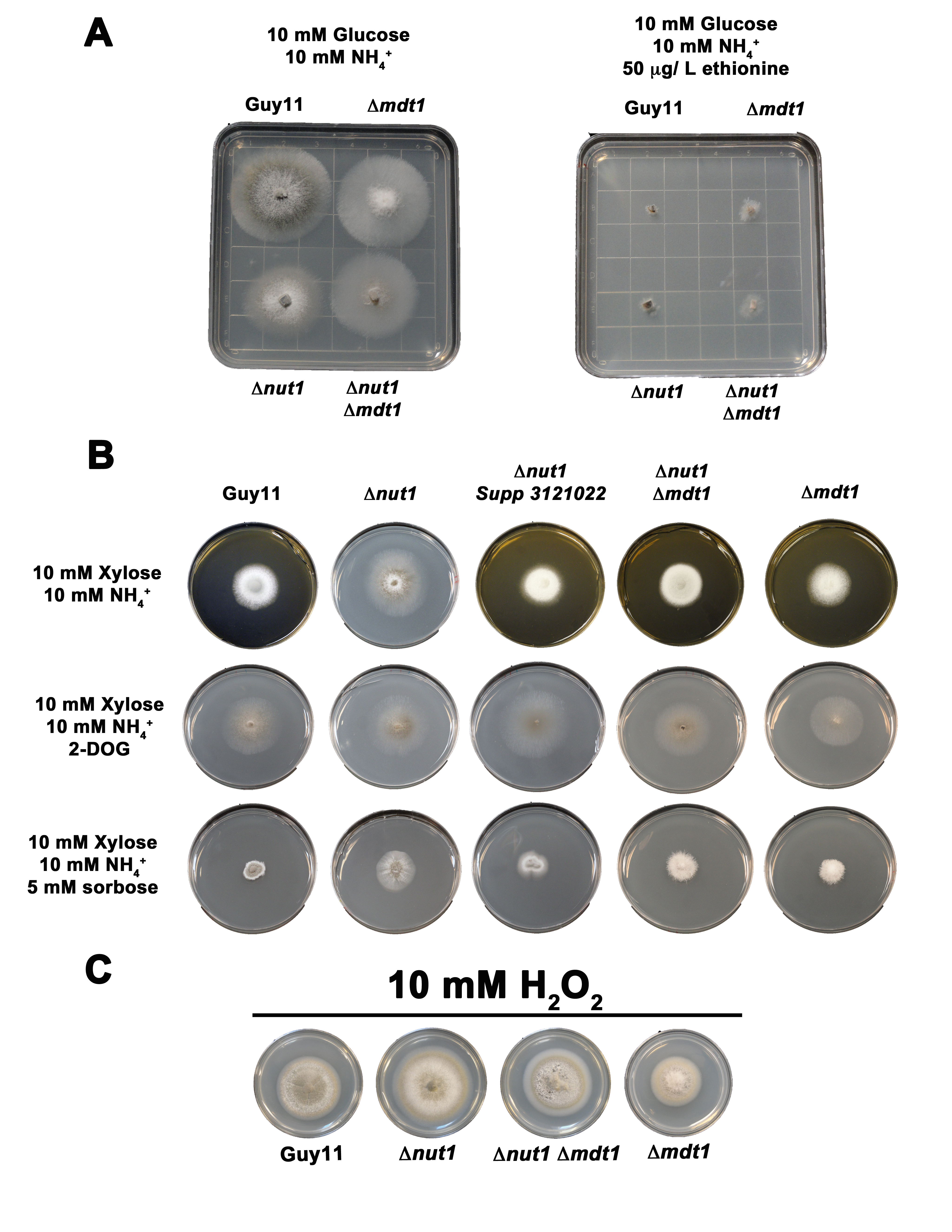

Supplement: Figure S9 — Exploring Mdt1 function. (A) To determine if the Mdt1 efflux protein has a role in ethionine resistance, strains were grown on minimal media with 10 mM glucose and 10 mM NH4 + (left panel) or the same media supplemented with 50 µg/L ethionine. Even at the relatively high concentrations of ethionine shown, some growth was observed for MDT1 deletion strains, suggesting deletion of MDT1 did not make M. oryzae more susceptible to ethionine. (B) To determine if loss of Mdt1 function renders strains defective in glucose uptake, Guy11, Δnut1, dnut1 Supp 3121022, Δnut1 Δmdt1 and Δmdt11 strains were grown for 10 days on 85 mm petri-dishes containing carbon derepressing minimal media consisting of 10 mM xylose+10 mM NH4 + as sole carbon and nitrogen sources and the same media supplemented with 5 mM sorbose or 50 µg/mL 2-deoxyglucose (2-DOG). Strains with disrupted Mdt1 function were not more resistant to sorbose or 2-DOG compared to Guy11, suggesting glucose uptake and/or phosphorylation is not significantly impaired in these strains. (C) Mdt1 does not confer resistance to reactive oxygen species. Strains were grown for 10 days on CM supplemented with 10 mM H2O2. (TIF) [file pgen.1002673.s009.tif]

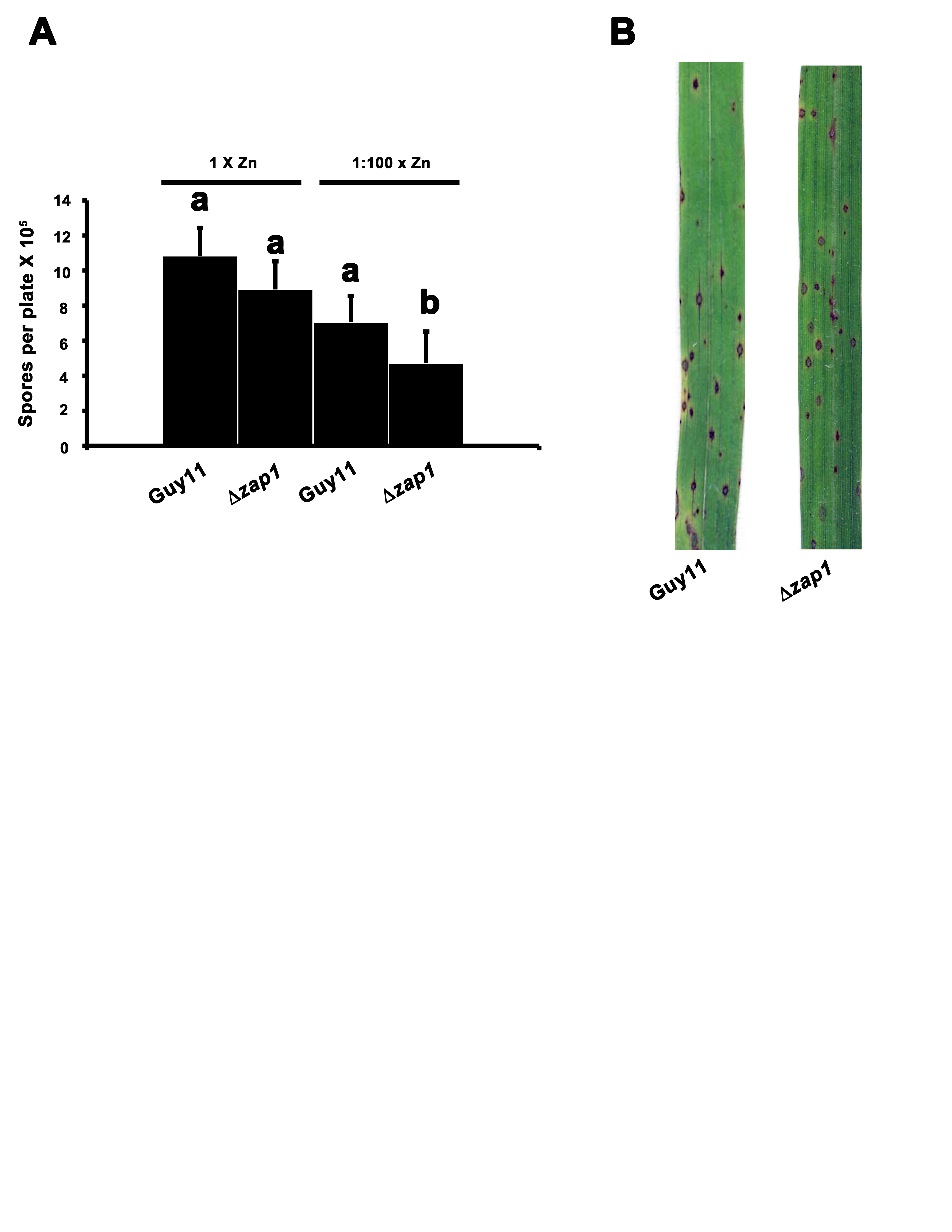

Supplement: Figure S10 — The role of zinc metabolism in rice blast disease. (A) ZAP1 encodes a putative zinc finger protein with a role in zinc acquisition. Guy11 and Δzap1 strains were grown on minimal media containing the standard zinc concentration (1×Zn) or 100-fold less zinc (1∶100×Zn). Reduced sporulation of Δzap1 on 1∶100×Zn GMM indicates ZAP1 has a role in zinc homeostasis and acquisition. Spores were harvested from plates following 12 days of growth. Values are the average of at least three independent replicates and bars are standard error. Bars with the same letters are not significantly different (Student's t-test p≤0.05). (B) ZAP1 is not required for infection. Guy11 and Δzap1 strains were inoculated at a rate of 1×10^5^ spores/ml. No loss of pathogenicity was observed in Δzap1 strains indicating reduced zinc uptake from the plant is not an impediment to disease establishment. (TIF) [file pgen.1002673.s010.tif]
