# Supplementary material for: Principles of Carbon Catabolite Repression in the Rice Blast Fungus: Tps1, Nmr1-3, and a MATE–Family Pump Regulate Glucose Metabolism during Infection
Source: PLoS Genet. 2012 May 3;8(5):e1002673. doi: 10.1371/journal.pgen.1002673 (PMC3342947; doi:10.1371/journal.pgen.1002673)
Supplement: Table S1 — Description of Magnaporthe oryzae genes analysed in this study. (DOC) [file pgen.1002673.s011.doc]

**Table S1**. Description of *Magnaporthe oryzae* genes analysed in this study.

| **Locus** | ***M. oryzae* Gene Name** | **Reference** | **Molecular function** | **Function reference** |
| --- | --- | --- | --- | --- |
| MGG_00040 | *GHT2* | 1 | ^a^ High affinity glucose transporter | 2 |
| MGG_01446 | *RGT2* | 1 | ^a^ High affinity glucose transporter | 3 |
| MGG_10508 | *HXT1* | 1 | ^a^ Low affinity glucose transporter | 4 |
| MGG_09289 | *HXK1* | 1 | Hexokinase 2 | 5 |
| MGG_00623 | *HXK2* | 1 | ^a^ Hexokinase 1 | 6 |
| MGG_03041 | *GLK1* | 1 | ^a^ Glucokinase | 7 |
| MGG_00189 | *PRN3* | 1 | ^a^ Δ-1-pyrroline-5-carboxylate dehydrogenase | 8 |
| MGG_00625 | *GNI1* | 1 | ^a^ Glucosamine 6 phosphate isomerase/deaminase | 9 |
| MGG_01404 | *XYR1* | 1 | ^a^ NAD(P)H-dependent D-xylose reductase | 10 |
| MGG_03123 | *MDT1* | 1 | MATE-family efflux pump | *This study* |
| MGG_03880 | *ADH1* | 1 | ^a^Alcohol dehydrogenase | 11 |
| MGG_04895 | *ICL1* | 1 | Isocitrate lyase | 12 |
| MGG_02653 | *PFK1* | 1 | ^a^6-phosphofructokinase | 13 |
| MGG_08895 | *FBP1* | 1 | ^a^Fructose-1,6-bisphosphatase | 14 |
| MGG_03670 | *SPM1* | 1 | Vacuolar serine protease | 15 |
| MGG_05871 | *PTH11* | 1 | Integral membrane protein | 16 |
| MGG_09272 | *ß-glucosidase 1* | 1 | ^a^Cell wall degrading enzyme | 17 |
| MGG_05529 | *feruloyl esterase B* | 1 | ^a^Cell wall degrading enzyme | 17 |
| MGG_10712 | *exoglucanase 1* | 1 | ^a^Cell wall degrading enzyme | 17 |
| MGG_06062 | *NIA1* | 1 | Nitrate reductase | 18 |
| MGG_09926 | *G6PDH* | 1 | Glucose-6-phosphate dehydrogenase | 18 |
| MGG_00604 | *TUB2* | 1 | Tubulin β chain | 19 |
| MGG_03982 | *ACT1* | 1 | Actin | 20 |

^a^ Putative function based on sequence homology.

1. Dean RA, Talbot NJ, Ebbole DJ, Farman ML, Mitchell TK, et al. (2005) The genome sequence of the rice blast fungus *Magnaporthe grisea.* Nature 434: 980-986.

2. Heiland S, Radovanovic N, Hofer M, Windrickx J, Lichtenberg H (2000) Multiple hexose transporters of *Schizosaccharomyces* *pombe*. J Bact 182: 2153 – 2162.

3. Ozcan S, Dover J, Rosenwald AG, Wölfl S, Johnston M (1996) Two glucose transporters in *Saccharomyces cerevisiae* are glucose sensors that generate a signal for induction of gene expression. Proc Natl Acad Sci U S A 93:12428 - 12432.

4. Lewis DA, Bisson LF (1991) The HXT1 gene product of *Saccharomyces cerevisiae* is a new member of the family of hexose transporters. Mol Cell Biol 11: 3804-3813.

5. Wilson RA, Jenkinson JM, Gibson RP, Littlechild JA, Wang, ZY, et al. (2007) Tps1 regulates the pentose phosphate pathway, nitrogen metabolism and fungal virulence. EMBO J 26: 3673-3685.

6. Rodríguez A, De La Cera T, Herrero P, Moreno F (2001) The hexokinase 2 protein regulates the expression of the GLK1, HXK1 and HXK2 genes of *Saccharomyces cerevisiae*. Biochem J 355: 625-631.

7. Clifton D, Walsh RB, Fraenkel DG (1993) Functional studies of yeast glucokinase. J Bacteriol 175: 3289 - 3294.

8. Gómez D, García I, Scazzocchio C, Cubero B (2003) Multiple GATA sites: protein binding and physiological relevance for the regulation of the proline transporter gene of *Aspergillus nidulans*. Mol Microbiol 50: 277 - 289.

9. Comb DG, Roseman S (1958) Glucosamine metabolism. IV. Glucosamine-6-phosphate deaminase. J Biol Chem 232: 807 – 827.

10. Lee H (1998) The structure and function of yeast xylose (aldose) reductases. Yeast 14: 977 - 984.

11. Pateman JA, Doy CH, Olsen, JE, U. Norris U, Creaser EH, Hynes M (1983) Regulation of Alcohol Dehydrogenase (ADH) and Aldehyde Dehydrogenase (ALDDH) in Aspergillus nidulans. Proc R Soc Lond B 217: 243 - 264.

12. Wang ZY, Thornton CR, Kershaw MJ, Debao L, Talbot NJ (2003) The glyoxylate cycle is required for temporal regulation of virulence by the plant pathogenic fungus *Magnaporthe grisea*. Mol Microbiol 47: 1601 - 12.

13. Evans PR, Hellinga HW (1987) Mutations in the active site of *Escherichia coli* phosphofructokinase. Nature 327: 437 - 439.

14. Marcus F, Gontero B, Harrsch PB, Rittenhouse J (March 1986) Amino acid sequence homology among fructose-1,6-bisphosphatases. Biochem Biophys Res Commun 135: 374 – 81.

15. Donofrio NM, Oh Y, Lundy R, Pan H, Brown DE, Jeong JS, Coughlan S, Mitchell TK, Dean RA (2006) Global gene expression during nitrogen starvation in the rice blast fungus, Magnaporthe grisea. Fungal Genet Biol 43: 605 – 617.

16. DeZwaan TM, Carroll AM, Valent B, Sweigard JA (1999) *Magnaporthe grisea* pth11p is a novel plasma membrane protein that mediates appressorium differentiation in response to inductive substrate cues. Plant Cell 11: 2013 - 2030.

17. Walton, J.D (1994) Deconstructing the cell wall. Plant Physiol. 104: 1113–1118.

18. Wilson, RA, Gibson, RP, Quispe, CF, Littlechild, JA and Talbot, NJ (2010) An NADPH-dependent genetic switch regulates plant infection by the rice blast fungus. *Proc Natl Acad Sci USA* 107: 21902 – 21907.

19. Nogales E, Wolf SG, Downing KH (1998) Structure of the alpha-beta tubulin dimer by electron crystallography. Nature 391:199 - 203.

20. Mosquera G, Giraldo MC, Khang CH, Coughlan S, Valent B (2009) Interaction transcriptome analysis identifies *Magnaporthe oryzae* BAS1-4 as Biotrophy-associated secreted proteins in rice blast disease. *Plant Cell* 21: 1273 - 1290.
