# Supplementary material for: Principles of Carbon Catabolite Repression in the Rice Blast Fungus: Tps1, Nmr1-3, and a MATE–Family Pump Regulate Glucose Metabolism during Infection
Source: PLoS Genet. 2012 May 3;8(5):e1002673. doi: 10.1371/journal.pgen.1002673 (PMC3342947; doi:10.1371/journal.pgen.1002673)
Supplement: Table S3 — Extragenic suppressors of Δnut1 strains generated by Agrobacterium tumefaciens-mediated mutagenesis. (DOCX) [file pgen.1002673.s013.docx]

**Table S3**. Extragenic suppressors of Δ*nut1* strains generated by *Agrobacterium tumefaciens*-mediated mutagenesis.

| **Selection Media** | **Parental strain** | **Suppressor strain** | **Locus** | **Gene** | **Molecular function** |
| --- | --- | --- | --- | --- | --- |
| 10 mM Glucose 10 mM Proline | Δ*nut1* | Δ*nut1 Supp 3121022* | MGG_03123 (1) | *MDT1* | Multidrug and toxin extrusion (MATE) protein-1 (2) |
| 10 mM Glucose 10 mM Proline | Δ*nut1* | Δ*nut1 Supp 3121023* | MGG_03123 (1) | *MDT1* | Multidrug and toxin extrusion (MATE) protein-1 (2) |
| 10 mM Glucose 10 mM Proline | Δ*nut1* | Δ*nut1 Supp 3121025* | MGG_03123 (1) | *MDT1* | Multidrug and toxin extrusion (MATE) protein-1 (2) |
| 10 mM Glucose 10mM Glucosamine | Δ*nut1* | Δ*nut1 Supp 3121042* | MGG_03123 (1) | *MDT1* | Multidrug and toxin extrusion (MATE) protein-1 (2) |

1. Dean RA, Talbot NJ, Ebbole DJ, Farman ML, Mitchell TK, et al. (2005) The genome sequence of the rice blast fungus *Magnaporthe grisea.* Nature 434: 980-986.

2. Brown MH, Paulsen IT, Skurray RA (1999) The multidrug efflux protein NorM is a prototype of a new family of transporters. Mol Microbiol 31: 394–395.
