# Supplementary material for: Principles of Carbon Catabolite Repression in the Rice Blast Fungus: Tps1, Nmr1-3, and a MATE–Family Pump Regulate Glucose Metabolism during Infection
Source: PLoS Genet. 2012 May 3;8(5):e1002673. doi: 10.1371/journal.pgen.1002673 (PMC3342947; doi:10.1371/journal.pgen.1002673)
Supplement: Table S4 — Magnaporthe oryzae strains used in this study. (DOCX) [file pgen.1002673.s014.docx]

**Table S4**. *Magnaporthe oryzae* strains used in this study.

| **Strains** | **Genotype** | **Reference** |
| --- | --- | --- |
| Guy11 | Wild type | 1 |
| Δ*tps1* | Trehalose-6-phosphate synthase 1 deletion mutant of Guy11 | 2 – 5 |
| Δ*nut1* | Nitrogen metabolite repression mutant of Guy1 | 4; *This study* |
| Δ*tps1* Δ*nmr1* | NmrA-like family member deletion mutant of Δ*tps1* | 4 |
| Δ*tps1* Δ*nmr2* | NmrA-like family member deletion mutant of Δ*tps1* | 4 |
| Δ*tps1* Δ*nmr3* | NmrA-like family member deletion mutant of Δ*tps1* | 4 |
| Δ*nmr1* Δ*nmr2* Δ*nmr3* | Triple NmrA-like family member deletion mutant of Guy11 | 4 |
| Δ*tps1::R22G* | Complementation of Δ*tps1* with *TPS1* gene variant encoding the amino acid substitution R22G | 3 |
| Δ*tps1::Y99V* | Complementation of Δ*tps1* with *TPS1* gene variant encoding the amino acid substitution Y99V | 3 |
| Δ*tps1* Δ*nut1* | Trehalose-6-phosphate synthase 1 deletion mutant of Δ*nut1* | *This study* |
| Δ*nut1* *3121022* | Extragenic suppressor of Δ*nut1* resulting from *Agrobacterium tumefaciens*-mediated disruption of *MDT1* | *This study* |
| Δ*nut1* Δ*mdt1* | MATE-family efflux protein deletion mutant of Δ*nut1* | *This study* |
| Δ*mdt1* | MATE-family efflux protein deletion mutant of Guy11 | *This study* |
| Δ*mdt1 MDT1* | Complementation strain of Δ*mdt1* resulting from integration of full length *MDT1* gene and native promoter into the genome of strains carrying the Δ*mdt1* gene deletion. | *This study* |
| Δ*tps1* Δ*mdt1* | MATE-family efflux protein deletion mutant of Δ*tps1* | *This study* |
| Δ*hxk1* | Deletion mutant of yeast *Hxk2* homologue | 3; *This study* |
| Δ*hxk2* | Deletion mutant of yeast *Hxk1* homologue | *This study* |
| Δ*glk1* | Deletion mutant of glucokinase 1 | 6; *This study* |
| Δ*zap1* | Zinc-responsive Activator Protein deletion mutant of Guy11 | *This study* |

1. Talbot, NJ (2003) On the Trail of a Cereal Killer: Exploring the Biology of Magnaporthe grisea. Ann Rev Micro 57: 177 - 202

2. Foster AJ, Jenkinson JM, Talbot NJ (2003) Trehalose synthesis and metabolism are required at different stages of plant infection by *Magnaporthe grisea*. EMBO J 22: 225 - 235.

3. Wilson RA, Jenkinson JM, Gibson RP, Littlechild JA, Wang Z-Y, Talbot NJ (2007) Tps1 regulates the pentose phosphate pathway, nitrogen metabolism and fungal virulence. EMBO J 26: 3673-3685.

4. Wilson RA, Gibson RP, Quispe CF, Littlechild JA, Talbot NJ (2010) An NADPH-dependent genetic switch regulates plant infection by the rice blast fungus. Proc Natl Acad Sci USA 107: 21902 - 21907.

5. Fernandez J, Wilson RA (2011) The sugar sensor, trehalose-6-phosphate synthase (Tps1), regulates primary and secondary metabolism during infection by the rice blast fungus: Will *Magnaporthe oryzae*ʼs “sweet tooth” become its “Achillesʼ heel”? Mycology 2: 46 - 53.

6. Zhang L, Lv R, Dou X, Qi Z, Hua C, Zhang H et al (2011) The function of MoGlk1 in integration of glucose and ammonium utilization in *Magnaporthe oryzae*. PLoS One 6: e22809.
