# Supplementary material for: Principles of Carbon Catabolite Repression in the Rice Blast Fungus: Tps1, Nmr1-3, and a MATE–Family Pump Regulate Glucose Metabolism during Infection
Source: PLoS Genet. 2012 May 3;8(5):e1002673. doi: 10.1371/journal.pgen.1002673 (PMC3342947; doi:10.1371/journal.pgen.1002673)
Supplement: Table S5 — Oligonucleotide primers used in this study. (DOC) [file pgen.1002673.s015.doc]

**Table S5**. Oligonucleotide primers used in this study.

| **Gene** | **Primer** | **Sequence 5’ – 3’** |
| --- | --- | --- |
| *ILV1^a^* | M13F:IL^c^ | ^d^**CGCCAGGGGTTTTCCCAGTCACGAC**GTCGACGTGCCAACGCCACAG |
|  | ILSplit^c^ | AAGCATGTGCAGTGCCTTC |
|  | M13R:LV1^c^ | ^e^**AGCGGATAACAATTTCACACAGGA**GTCGACGTGAGAGCATGCTAA |
|  | LV1Split^c^ | CGCCCGGCCGACATCC |
| *bar*^b^ | M13F:BA^c^ | ^d^**CGCCAGGGGTTTTCCCAGTCACGAC**GTCGACAGAAGATGATATTGAAGGAG |
|  | BaSplit^c^ | GAGCCCAGTCCCGTCCG |
|  | M13R:AR^c^ | ^e^**AGCGGATAACAATTTCACACAGGA**CTAAATCTCGGTGACGGGCAGG |
|  | ArSplit^c^ | CGCCCGGCCGACATCC |
| *NUT1^f^* | LF5’ | GCTGTGAATGATTCTCCCCCTTCC |
|  | LF3’ | ^d^**GTCGTGACTGGGAAAACCCTGGCG**TGTTGCGGCTGGATCCTTTATTC |
|  | RF5’ | ^e^**TCCTGTGTGAAATTGTTATCCGCT**ACTTCTCCCCCAAAACAACAGG |
|  | RF3’ | CGGATAGACTAGATGTTTTTTTTTCTTTCTTT |
|  | NesF | CTCCGGCCGTGAATGAATTGTG |
|  | NesR | TCCCCCTTCTTCACTTCTTTTCCTAG |
| *HXK1^f^* | LF5’ | GAACAGAACATCATGGCGGTTGG |
|  | LF3’ | ^d^**GTCGTGACTGGGAAAACCCTGGCG**GTGCCTGTTCAGTATTCTCAGTCAGTTTC |
|  | RF5’ | ^e^**TCCTGTGTGAAATTGTTATCCGCT**GACCCAAAGAAGATTGAGAAGCTGC |
|  | RF3’ | CTTTGCAGGCCAGTCGAGGATC |
|  | NesF | GGTCACTCAAGGGCAACTTTCTTTACT |
|  | NesR | CCTGAGGGCCTGGGCACC |
| *HXK2^f^* | LF5’ | GTACTGGTAGTTTATGTATAGGTAGGTAGGCACTC |
|  | LF3’ | ^d^**GTCGTGACTGGGAAAACCCTGGCG**GGCTGCATGACACCGGGAGAC |
|  | RF5’ | ^e^**TCCTGTGTGAAATTGTTATCCGCT**GAAACACTCAAATCGGCGTCAGAG |
|  | RF3’ | GACGCGACCGAGATCCAGCTC |
|  | NesF | GTAACAGGACTAGACTAGGCTTCATTGCTC |
|  | NesR | GCTCTAGTCTCCTGTGCGAATGGC |
| *GLK1 ^f^* | LF5’ | GAGCCCAGTCAAACGTTGTATTCG |
|  | LF3’ | ^d^**GTCGTGACTGGGAAAACCCTGGCG**CTTGAGGGGTCTCGAGGTCGG |
|  | RF5’ | ^e^**TCCTGTGTGAAATTGTTATCCGCT**GACAGTATTGGAAGTCATGAGCGCG |
|  | RF3’ | GGAGGTGGTGCTGTGCAGACG |
|  | NesF | CTTCGCCTAGCCGTGCCG |
|  | NesR | GCGCCACATATTCCCCGTACC |
| *MDT1^f^* | LF5’ | CCCGAACCCACAGGAGCTACAA |
|  | LF3’ | ^d^**GTCGTGACTGGGAAAACCCTGGCG**GAAAAAGACAACATAAAGCACCAGCAG |
|  | RF5’ | ^e^**TCCTGTGTGAAATTGTTATCCGCT**GCCTCCAAAACCATGATCGTCTG |
|  | RF3’ | AAAGTTAGGTGATCTCATTGGAGCCC |
|  | NesF | GCCAACTCGAACGATATGCTGACT |
|  | NesR | GCCCCAAAGCACCTACAGCCT |
| *GHT2^g^* | qGHT2-F | CATCAACCGCCGCACCAAG |
|  | qGHT2-R | CTTGGCGTGGTCGTGGTTGA |
| *RGT2 ^g^* | qRGT2-F | GCTCGCCTTCGCCTACTTCCT |
|  | qRGT2-R | TCCTCCTTGGCAGACTCGGTG |
| *HXT1 ^g^* | qhxt1-F | TTGCTTGGTTATTTGACGGGGAG |
|  | qhxt1-R | GACCTTCTTCTCATCAGCCACGC |
| *HXK1 ^g^* | QRT-Hxk1 F | GAACTACCGCTCTTGCCATGTGG |
|  | QRT-HXK1 R | TCACTTAAAGTTCTCTGGGTTGCTGATG |
| *HXK2 ^g^* | QRT-Hxk2 F | CAGGAGACTAGAGCCGAGCTGGAT |
|  | QRT-Hxk2 R | TTCAAGACACGCCAGCGCC |
| *GLK1 ^g^* | qGLK1-F | GCTGTGGTCCTGAAGACGGGC |
|  | qGLK1-R | GCACTCTTCTCCATCTCAGCCG |
| *PRN3 ^g^* | qPRN3-F | CTACGATGACGACCTTCACGGC |
|  | qPRN3-R | TCCACTCGCCCTGCCACC |
| *GNI1 ^g^* | Gni1 qPCR-F | CGTCTCGCCGGACCTGGTTC |
|  | Gni1 qPCR-R | ACAAGCTGGGTCGAGACAAACG |
| *XYR1 ^g^* | qXR1-F | CCCTCTTCACCCACCCCACC |
|  | qXR1-R | GATGTAGATGGGAGGGCTCAGGTC |
| *MDT1 ^g^* | RT-Mat1 F1 | GCCAACTCGAACGATATGCTGACT |
|  | RT-Mat1 R1 | GCCCCAAAGCACCTACAGCCT |
| *TUB2 ^g^* | QRT-PCR b-tub F2 | CGCGGCCTCAAGATGTCGT |
|  | QRT-PCR b-tub R2 | GCCTCCTCCTCGTACTCCTCTTCC |
| *ADH1 ^g^* | qADH-F | ATCACCATCAAGGGCAGCTACG |
|  | qADH-R | TCCCTACGACCTTGCCCTGCT |
| *ICL1 ^g^* | qICL1-F | AGAGCAACAGGGAAGCCAGAGC |
|  | qICL1-R | GCTGCTCACACCACCCGTCA |
| *PFK1 ^g^* | qPFK-F | CCGATGAAGGAGGTGGAGGACA |
|  | qPFK-R | CAGTCCACGTTTGACATCTTTAGCC |
| *FBP1 ^g^* | qFBP-F | CCGCCCCTAACCTTTTGAAATACT |
|  | qFBP-R | CTTTACTTTTTCAACCTCATCCCAGC |
| *SPM1 ^g^* | MGG_03670R | TCTTCGACTCTGTGAACGATGCCT |
|  | MGG_03670F | GTGAGGATGAGAGACTTGAGCT |
| *PTH11 ^g^* | QRT-PCR Pth11F1 | GCGTCCGACGTTCCCGAC |
|  | QRT-PCR Pth11R1 | GCTGAGAACGTCGATGGGGATCT |
| *ß-glucosidase 1 ^g^* | CWDE1 F1 | GACCCGCCCGTCGTCCT |
|  | CWDE1 R1 | GTTCAGGTCAGCACTCAGATGGAGC |
| *feruloyl esterase B ^g^* | CWDE2 F1 | CCTATGACCGCGTCGCCG |
|  | CWDE2 R1 | CTACCCCATTCCACTTTGACCTGTGC |
| *exoglucanase 1 ^g^* | CWDE3 F1 | CTAAGCTCAACGCCGCCTACG |
|  | CWDE3 R1 | CCAATGGGGCCGAACCG |
| *NIA1 ^g^* | Nia1F1 | TGGCAACCGAACAGAGGAAGAC |
|  | Nia1R1 | TCAGAAAAACAACAAATCATCATCCTT |
| *G6PDH ^g^* | G6PDHF1 | GATCCCTGAGGCTTACGAATCATTGA |
|  | G6PDHR1 | TCAGAACTTGTTAGCGTGCAGCG |
| *ACT1^g^* | MgActinF | AGCGTGGTATCCTCACTTTGCG |
|  | MgActinR | TCATCTTCTCTCGGTTGGACTTGG |
| *Hph* ^h^ | Hyg 5’ Fw ^i^ | GAAAAAGCCTGAACTCACCGCG |
|  | Hyg 5’ Rev ^i^ | CGCGGTGAGTTCAGGCTTTTTC |
|  | Hyg 3’ Fw ^i^ | ACCGACGCCCCAGCACTC |
|  | HYG 3’ Rev ^i^ | GAGTGCTGGGGCGTCGGT |
| *MDT1* | MAT1-GF^J^ | ^k^TATAGGGCGAATTGGGTACTCAAATTGGTTGTGATTTCAAACCATGCGAACCTT |
|  | MAT1-GR^J^ | ^k^CCCGGTGAACAGCTCCTCGCCCTTGCTCACCTGACACTGCCATTGCCAATATTG |

^a^ Encoding the sulphonylurea resistance cassette [1]. ^b^ Encoding the bialaphos resistance marker [2]. ^c^ Primers used to amplify the selectable marker for split marker deletion ([3] and Figure S5). ^d^ M13F sequence, highlighted in bold, is upstream of the gene specific sequence [3]. ^e^ M13R sequence, highlighted in bold, is upstream of the gene specific sequence [3]. ^f^ Primers for splitmarker deletion construct [3]. ^g^ Primers used for qRT-PCR analysis. ^h^ Encoding the hygromycin resistance cassette [4]. ^I^ Primers for inverse PCR. ^J^Primers for Δ*mdt1* complementation. ^k^ Underlined sequence is homologous to the *XhoI* site flanking sequences on the yeast vector pDL2 [5].

1. Sweigard J, Chumley FG, Carroll A, Farrall L and Valent B (1997) A series of vectors for fungal transformation. *Fungal Genet Newsl* 44: 52-53.

2. Pall ML, Brunelli JP (1993) A series of six compact fungal transformation vectors containing polylinkers with multiple unique restriction sites. *Fungal Genet Newsl* 40: 59-63.

3. Wilson, RA, Gibson, RP, Quispe, CF, Littlechild, JA and Talbot, NJ (2010) An NADPH-dependent genetic switch regulates plant infection by the rice blast fungus. *Proc Natl Acad Sci USA* 107: 21902 – 21907.

4. Carroll AM, Sweigard JA, Valent B (1994) Improved vectors for selecting resistance to hygromycin. *Fungal Genet Newsl* 41: 22.

5. Zhou X, Li G, Xu J-R (2011) Efficient Approaches for Generating GFP Fusion and Epitope-Tagging Constructs in Filamentous Fungi*. Fungal Genomics* 722: 199-212. J.-R. Xu and B.H. Bluhm, editors. Humana Press.
